# ‘Risk-benefit’ assessment for comprehensive safety evaluation of Chinese patent medicines containing four common toxic ingredients: an analysis of clinical risk factors

**DOI:** 10.3389/fphar.2024.1324509

**Published:** 2024-08-23

**Authors:** Nan Zhang, Changming Zhong, Guoxiu Liu, Siyu Li, Li Lin, Namin Wei, Yu Song, Xiaoqing Wan, Yanping Wang, Yongyan Wang, Wanlin Wu, Zhongzhi Qian, Huaqiang Zhai

**Affiliations:** ^1^ Beijing University of Chinese Medicine, Beijing, China; ^2^ Wansheng Pharmaceutical Co. Ltd., Guizhou, China; ^3^ China Academy of Chinese Medical Sciences, Beijing, China; ^4^ Centre France Chine de la Médecine Chinoise, Seine, France; ^5^ Chinese Pharmacopoeia Commission, Beijing, China

**Keywords:** Chinese patent medicines containing toxic ingredients, clinical risk factors, TCM supervision, pharmacovigilanc, Chinese pharmacopoeia

## Abstract

**Background:**

Chinese patent medicines are specialty preparations in China that are produced using traditional prescriptions processed by modern pharmaceutical technology. They contain complex ingredients and much attention is paid to their clinical safety. Demonstrating the clinical safety of Chinese patent medicines containing toxic ingredients in modern pharmacological studies has become one of the urgent issues to be solved for the safe use of clinical medicines.

**Objectives:**

The aim of this research is to evaluate the safety of Chinese patent medicines containing toxic ingredients by applying the risk-benefit assessment method. Additionally, a database of ‘toxic ingredients–toxic Chinese herbal medicines-adverse reactions’ will be established to explore the relationship between toxic ingredients and adverse reactions. This will lay the foundation for the rational clinical use of Chinese patent medicines containing toxic ingredients.

**Methods:**

1) Establish a database of ‘toxic Chinese herbal medicines–toxic ingredients–toxic Chinese patent medicines’ to count the Chinese patent medicines containing toxic ingredients in the 2020 edition of *Chinese Pharmacopoeia*. 2) Filtered the clinical studies, extracted the drug-related ADEs, and analyzed the characteristics and correlations of these ADEs. 3) Finally, this section summarizes the causes of ADEs related to Chinese patent medicines containing toxic ingredients and extracts the main risk factors to provide a reference for further study.

**Outcomes:**

1) There are four main types of Chinese patent medicines containing toxic ingredients. These include medicines with diester aconitine metabolites, mineral composition, Araceae metabolites, and hydrogen cyanide. 2) Digestive system, skin and its appendages, and allergic reactions were the main types of ADEs related to four types of Chinese patent medicines containing toxic ingredients. 3) There are four primary risk factors associated with the clinical use of Chinese patent medicines containing toxic ingredients: medicine, medication, individual and regulatory factors.

## 1 Introduction

Risk-benefit assessment of medicines is an important consideration throughout the lifecycle of medicines. It is crucial in clinical research and development, marketing applications, and post-marketing regulatory decisions. This method has been widely used by scientists both domestically and internationally to evaluate the safety of medicines in clinical trials and provide guidance for their rational use in clinic settings.

The COVID-19 outbreak has had a significant impact on humanity, and traditional Chinese medicine (TCM) has played a crucial role in preventing and treating the epidemic in China and worldwide. This has resulted in greater global recognition of TCM (Bao et al., 2021). Consequently, there has been a greater focus on the safety of TCM, particularly its toxicity, which has been a subject of concern in previous studies, and has attracted widespread attention.

The National Center for Adverse Drug Reaction Monitoring (NCADRM) of China reported that from 2018 to 2020, TCM had 1.597 million, 1.635 million, and 1.798 million cases of adverse reactions/events (ADRs/ADEs), respectively. On average, TCM accounted for 13.6% of all ADRs/ADEs (National Center for Adverse Drug Reaction Monitoring, 2019; National Center for Adverse Drug Reaction Monitoring, 2020; National Center for Adverse Drug Reaction Monitoring, 2021). Toxicity research in TCM is considered a frontier issue in the ‘Major Scientific and Engineering Problems of Traditional Chinese Medicine in 2021’. (Tong et al., 2021). Currently, there is a lack of objective understanding among doctors, patients, and supervisors regarding the toxicity of Traditional Chinese Medicine (TCM). This has resulted in a phenomenon known as the ‘fear of toxicity’. The toxicity of TCM has been a topic of widespread controversy due to the kidney injury induced by aristolochic acid (AA) (Charen and Harbord, 2020). Multiple nations have banned AA-containing Chinese herbal medicines(CHMs), such as Manchurian Dutchmanspipe stem(*Isotrema manshuriense*), Aristolochia fangchi(*Isotrema fangchi*), and Dutchmanspipe root(*Aristolochia contorta Bunge*). Aristolochic acid analogues (AAAs) are a series of nitrophenanthrene carboxylic acid compounds widely found in Aristolochiaceae plants such as Aristolochia and Asarum. They are mainly divided into two categories: aristolochic acids (AAs) and aristolochic lactams (ALs). AAs include aristolochic acid A, B, C, D and so on. Aristolochic acid A is also known as aristolochic acid I (AA-I), and aristolochic acid B is also known as aristolochic acid II (AA-II). These two components are considered to be the most toxic aristolochic acid and can cause kidney damage. The toxicity of preparations containing aristolochic acids is closely related to its varieties, processing, compatibility, proportion in the compound prescription, preparation technology, clinical rational application and other links.

The toxicity of different AAAs varied greatly. AA-I had the strongest toxicity, followed by AA-II, AA-VIIIa, and AA-Ia, while other AAs and ALs showed no significant toxicity. At the same time, there are great differences in the types and contents of AAAs in different varieties of Aristolochiaceae Chinese medicine. At present, Manchurian Wildginger that is still in clinical use mainly contains AA-Ⅳa without obvious toxicity, and the toxic AA-Ⅰ and AA-Ⅱ contents are very low or not detected, so the risk is very low. Secondly, despite the fact that certain individual herbs contain elevated levels of toxic AAAs, including AA-Ⅰ, this does not necessarily imply that their compound preparations pose a risk. If the single herb accounts for a very low proportion in the total prescription, and at the same time, after the extraction and preparation process, the content of AA-Ⅰ in the final compound preparation may be very small, which will greatly reduce its risk. Finally, the toxicity of AAAS-containing CHMs was related to dose and duration of administration. Large doses of AAAs may cause severe acute renal failure. Even small doses of AAAs administration can lead to the accumulation of AAAs in the body if the long-term continuous use of Chinese medicine or Chinese patent medicine containing AAAs, which may lead to renal interstitial fibrosis. Therefore, the risk of AAAs-containing CHMs and their preparations does not depend on whether AAAs are contained, but on what kinds of AAAs are contained, whether they are toxic AAAs (such as AA-Ⅰ and AA-Ⅱ), and whether the content and dosage exceed the hazardous level. It shows that the toxicity of Chinese patent medicines(CPMs) containing toxic ingredients is not equal to the toxicity of medicinal materials. While strengthening the supervision of their production, we should also focus on the safety risks in the clinical application process.

TCM has a history of using medicinal substances that may have toxic effects. The toxicity of these substances has been defined in traditional pharmacological writings and standardised in the process of dispensing Chinese medicine. This not only involves processing drugs to reduce toxicity and increase efficacy but also regulating the decoction and use of drugs to achieve the clinical purpose of reducing drug toxicity, alleviating clinical adverse effects, and even using toxicity as a therapeutic approach in treatment. However, the limitations of ancient knowledge have made it unclear which ingredients of Chinese medicine are toxic and how they relate to clinical side effects and efficacy. This raises questions about the use of CPMs that contain toxic ingredients in today’s complex clinical drug environment. It is an urgent problem that needs to be addressed to ensure the safety of clinical drugs: demonstrating the clinical safety of CPMs containing toxic ingredients through modern pharmacological studies.

## 2 Overarching aims and objectives

Due to the complexity of toxic ingredients in CPMs and the diversity of Chinese medicine dispensing, we used the risk-benefit assessment method to comprehensively evaluate the safety of CPMs containing toxic ingredients. We created the ‘Toxic Ingredients–Toxic Chinese Medicine–Adverse Effects’ database by integrating major types and varieties of CPMs that contain toxic ingredients. We then analysed the characteristics of relevant ADEs to explore clinical safety risk factors. This lays the foundation for subsequent evaluation studies.

## 3 Material and methods

### 3.1 Database establishment of CPMs containing toxic ingredients in the 2020 edition of *Chinese Pharmacopoeia*

Based on the 2020 edition of *Chinese Pharmacopoeia*, this research presents a statistical analysis of CHMs with inherent properties of ‘small toxicity’, ‘toxicity’, and ‘strong toxicity’ in [nature, flavour and channel tropism] as well as CPMs containing these toxic CHMs. The CPMs have been classified based on the type of toxic ingredients they contain. A database of ‘toxic herb–toxic ingredient–toxic Chinese patent medicine’ has been constructed, providing a foundation for subsequent analysis.

**FIGURE 1:**
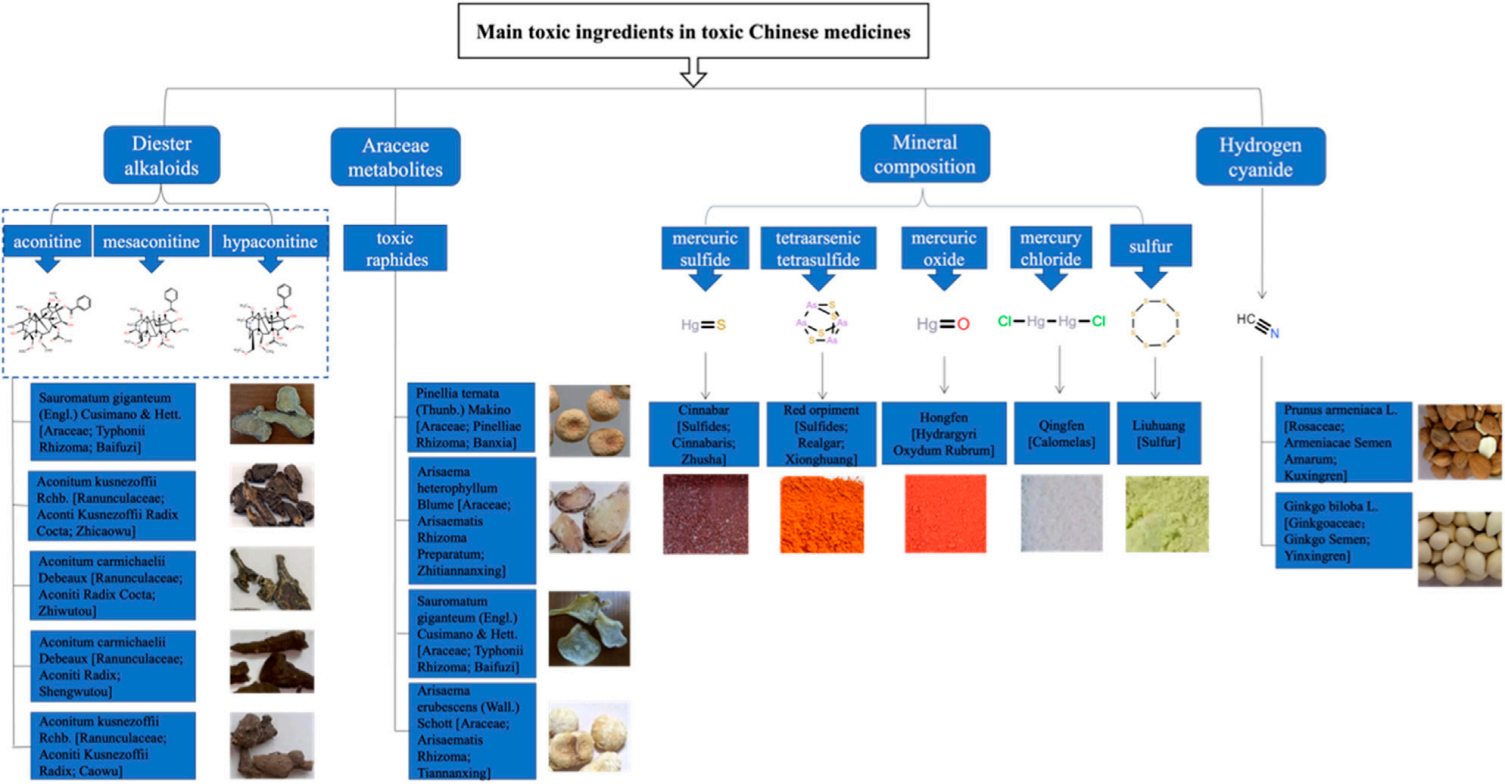
Main toxic ingredients and corresponding toxic CHMs selected in this study.

As the “Nature, Flavor, and Meridian Tropism” section of CHMs in the Chinese Pharmacopoeia describes their toxicity through the gradations of “small toxicity, toxicity, and strong toxicity,” the initial step was to collect these three categories of CHMs. Subsequently, CPMs containing these toxic CHMs were identified from the Pharmacopoeia, encompassing all CPMs containing toxic ingredients. These CPMs were then classified and counted. Given that the initial research focused on common types, CPMs containing toxic ingredients of lesser quantity were excluded, and those with a relatively higher quantity of toxic components were selected for further study (see Figure 1).

### 3.2 Collection of clinical researches and extraction of ADEs

#### 3.2.1 Inclusion and exclusion criteria

(1) Inclusion criteria

① Clinical trials and case reports, involving any type of disease; ② The interventions in the study (treatment group or control group) contain the name of the Chinese patent medicine in the database established above, without limitation of usage and dosage, and course of treatment; ③ The ADEs/ADRs are recorded in the study.

(2) Exclusion criteria

Exclusion criteria of research:

① Basic research, pharmacological research, pharmacodynamic research, and other research not involving clinical administration; ② Having the same name as the retrieved medicine but with a different formula; ③ The study without ADEs/ADRs recording; ④ Review research, scientific and technological achievements; ⑤ Duplicate publication.

Exclusion criteria of ADRs/ADEs:

① The description of ADRs/ADEs is unclear or erroneous; ② The retrieved medicine is irrelated with ADRs/ADEs, such as the retrieved medicine is not variable in the intervention measures or the formula of the retrieved medicine is added or subtracted.

The retrieved medicine exhibits no correlation with ADRs/ADEs, as evidenced by its name is identical to that mentioned in the literature, but the composition of the formula differs, or the formula of the retrieved medicine has undergone modifications.

#### 3.2.2 Literature retrieval

Using the names of CPMs containing toxic ingredients in the database established above as the keywords, we searched the China National Knowledge Infrastructure (CNKI) and Wanfang Database. The retrieval date is from the database record date to 29 August 2022.

## 4 Results

### 4.1 Database establishment of CPMs containing toxic ingredients in the 2020 edition of *Chinese Pharmacopoeia*

#### 4.1.1 General information on CPMs containing toxic ingredients

The statistical findings revealed that the Chinese Pharmacopoeia (2020) encompasses 83 distinct types of toxic CHMs. Among them, there are 10 CHMs with “strong toxicity” properties, 42 CHMs with “toxicity” properties, and 31 CHMs with “small toxicity” properties (see Supplementary Table S1). According to the above toxic CHMs, the varieties of CPMs containing these herbs were counted. There were 288 CPMs containing CHMs with “small toxicity” properties, 493 CPMs containing CHMs with “toxicity” properties, and 61 CPMs containing CHMs with “strong toxicity” properties. Some CPMs containing toxic ingredients contain 2 or more flavors of CHMs of different toxicity levels, so the number of corresponding CPMs was repeated in the statistical process. Therefore, the duplication items in the above CPMs were deleted, and the results showed that there were 523 CPMs containing toxic ingredients in the Chinese Pharmacopoeia (2020 edition).

#### 4.1.2 Classification of CPMs containing toxic ingredients

Toxic CHMs contain several toxic metabolites or compositions, including diester aconitine metabolites, mineral composition, Araceae metabolites, hyoscyamine, hydrogen cyanide, and cardiac glycoside. The CPMs were counted based on the types of toxic CHMs they contain, with the following order: Supplementary Table S1 shows the number and percentage of CPMs containing various ingredients. These include hydrogen cyanide (100, 26.67%), Araceae metabolites (92, 24.53%), mineral composition (87, 23.20%), diester aconitine metabolites (66, 17.60%), cardiac glycoside (21, 5.60%), and hyoscyamine (9, 2.40%) (see Supplementary Table S2). The percentage is the proportion of each class of Chinese patent medicine containing toxic ingredients in the total quantity of above six classes of Chinese patent medicine containing toxic ingredients.

**FIGURE 2:**
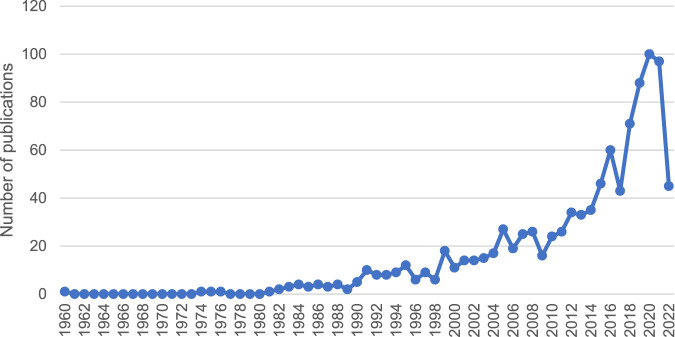
Trends in the Literature on ADEs of CPMs Containing Toxic ingredients.

This study focuses on four main types of CPMs that contain toxic ingredients: diester aconitine metabolites, mineral composition, Araceae metabolites, and hydrogen cyanide (see Supplementary Table S3). The diester aconitine metabolites consist of aconitine, mesaconitine, and hypaconitine. The mineral composition includes mercury, arsenic, and sulfur. Furthermore, the toxic metabolites of Araceae herbs are primarily toxic raphides containing lectin protein (see Figure 2).

### 4.2 Clinical research collection and ADE analysis

#### 4.2.1 Annual trends in clinical research publications

**FIGURE 3:**
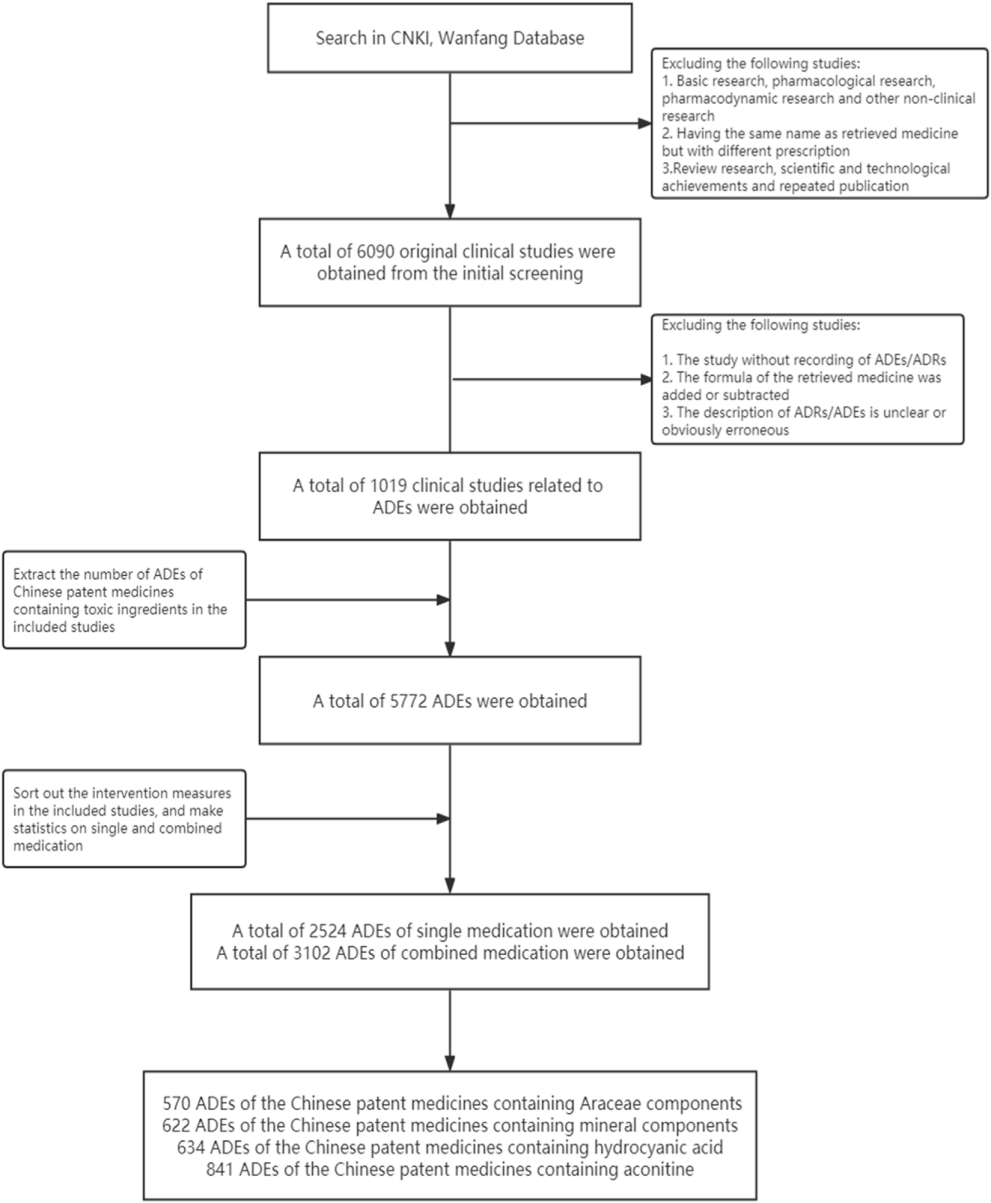
Literature screening and ADE extraction process.

After analysing 1,019 studies on ADEs caused by CPMs containing toxic ingredients, it is evident that the number of publications on this topic is increasing (see Figure 3). This indicates a growing concern for the safety of CPMs containing toxic ingredients.

#### 4.2.2 Literature screening and ADE extraction process

Based on the preliminary criteria, 6,090 original clinical studies of four types of CPMs were screened. Out of these, 1,019 studies involving ADEs were included in the study. These studies consisted of 336 CPMs containing Araceae metabolites, 246 CPMs containing hydrogen cyanide, 230 CPMs containing mineral composition, and 254 CPMs containing diester aconitine metabolites.

**FIGURE 4:**
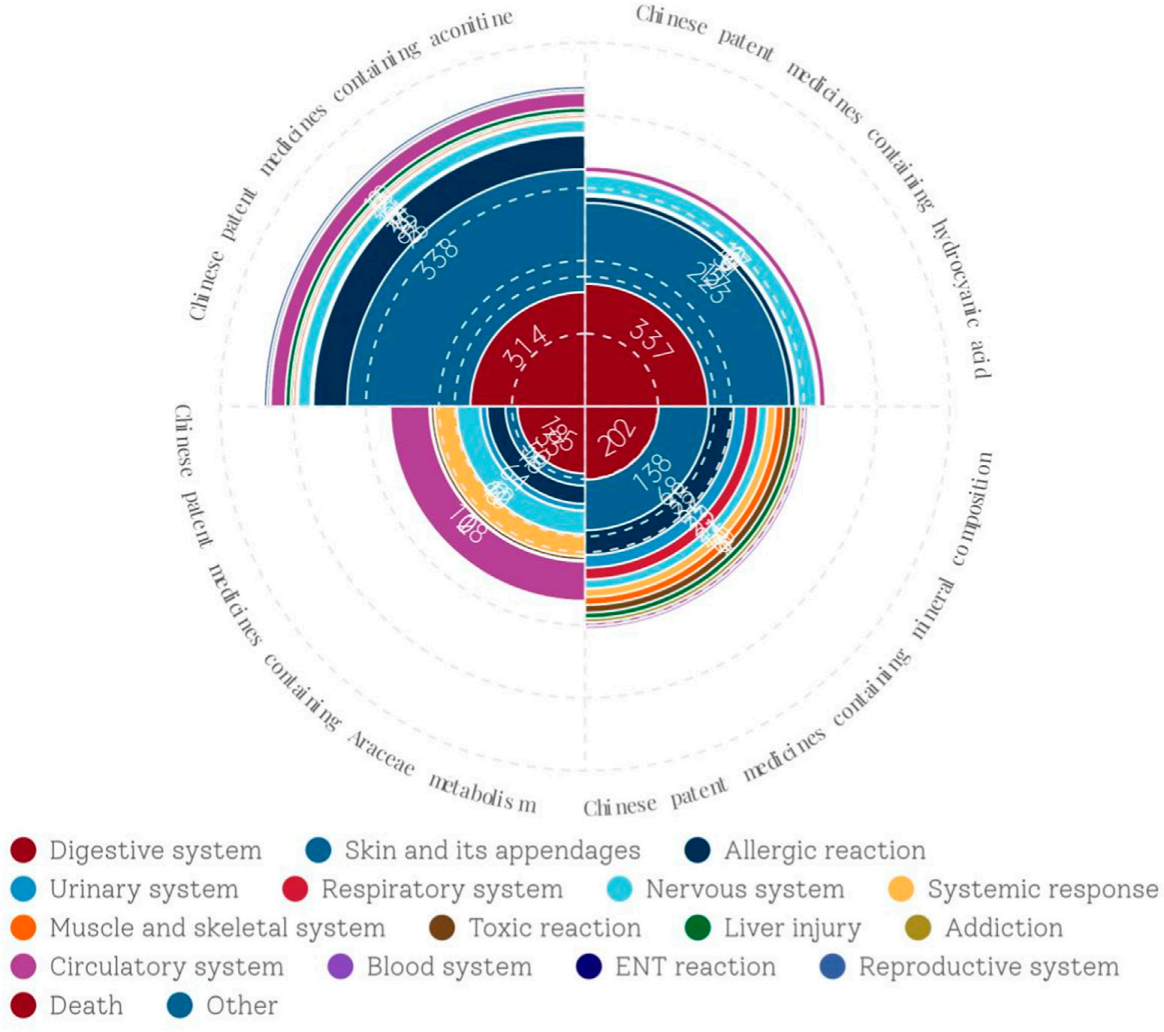
The ADEs involved in 4 kinds of CPMs containing toxic ingredients.

There were 5,772 ADEs associated with four types of CPMs. These included 1,673 cases of CPMs containing Araceae metabolites, 1,376 cases of CPMs containing hydrogen cyanide, 1,152 cases of CPMs containing mineral composition, and 1,762 cases of CPMs containing diester aconitine metabolites. There were 2,524 cases of ADEs related to single medication, 3,102 cases related to combined medication, and 146 cases with an unclear situation. In non-single medication studies, 111 cases of ADEs were related to the retrieved medicines or showed a significant increase in the combination group compared to the control group. The 111 ADEs mentioned above were combined with the ADEs related to the single medication, resulting in a total of 2,524 cases. Among these cases, 570 involved CPMs containing Araceae metabolites, 622 involved CPMs containing mineral composition, 634 involved CPMs containing hydrogen cyanide, and 841 involved CPMs containing diester aconitine metabolites (see Figure 4).

#### 4.2.3 Statistics and analysis of ADEs

(1) General information on the system/organ involved in the ADEs

**FIGURE 5:**
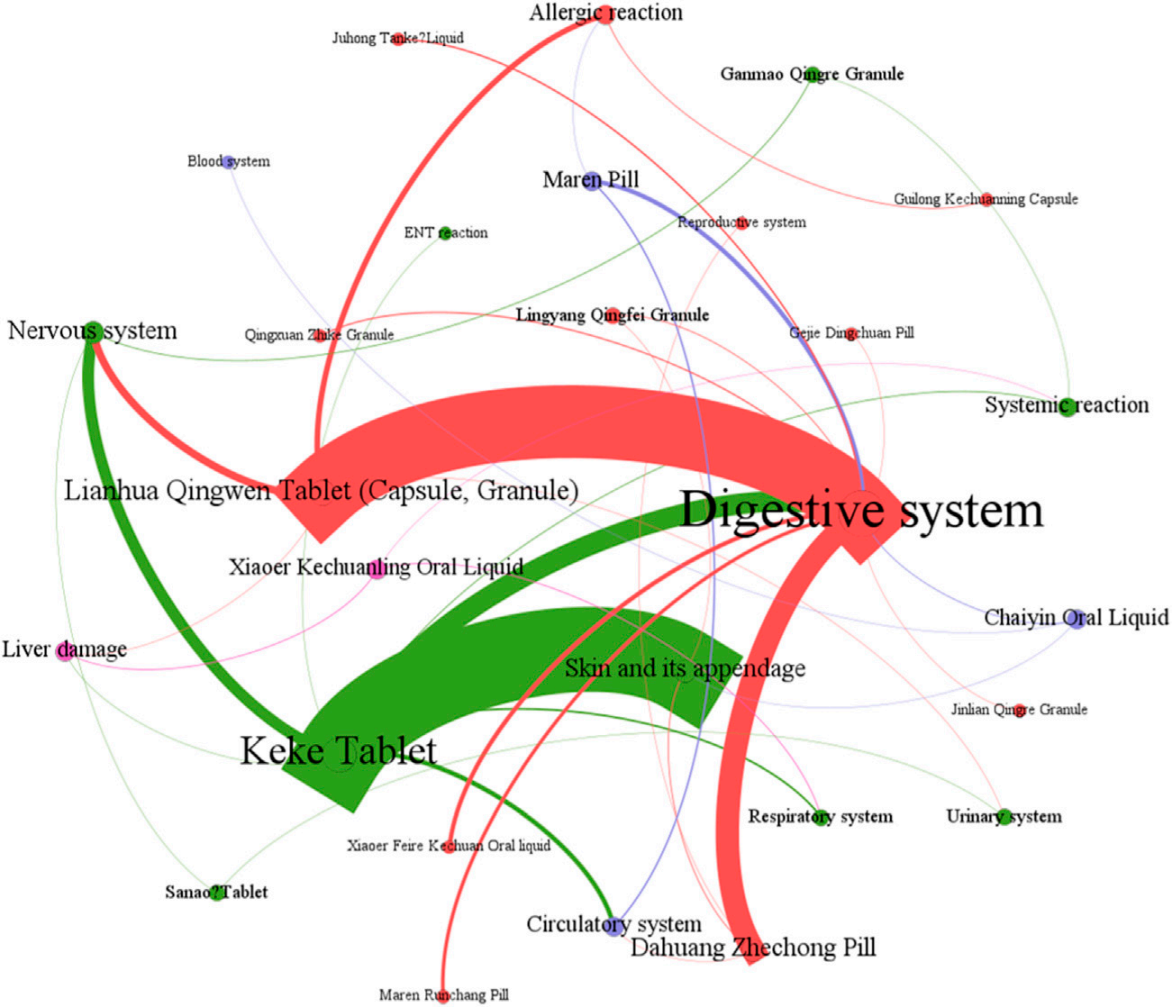
The network relationship between CPMs containing hydrogen cyanide and related ADEs.

Supplementary Table S4 classifies the ADEs related to CPMs containing toxic ingredients according to the involved system/organ. The results indicated that the digestive system was the most commonly affected system in CPMs containing hydrogen cyanide, followed by the skin and its appendages. The ADEs most commonly associated with CPMs containing Araceae metabolites are related to the digestive system, followed by the circulatory system, nervous system, systemic reaction, and allergic reaction. Similarly, the most common ADEs associated with CPMs containing mineral composition are still related to the digestive system, followed by the skin and its appendages and allergic reaction. The most common ADEs associated with CPMs containing diester aconitine metabolites involve skin and its appendages, followed by the digestive system, allergic reaction, circulatory system, and nervous system. In general, the main types of ADEs related to CPMs containing toxic ingredients are those affecting the digestive system, skin and its appendages, and allergic reaction, with the digestive system being the most frequently affected. Furthermore, Araceae-containing CPMs have a higher incidence of ADEs in the circulatory system, nervous system, and systemic reaction compared to the other three types (see Figure 5).

(2) Analysis of ADEs related to CPMs containing hydrogen cyanide

**FIGURE 6:**
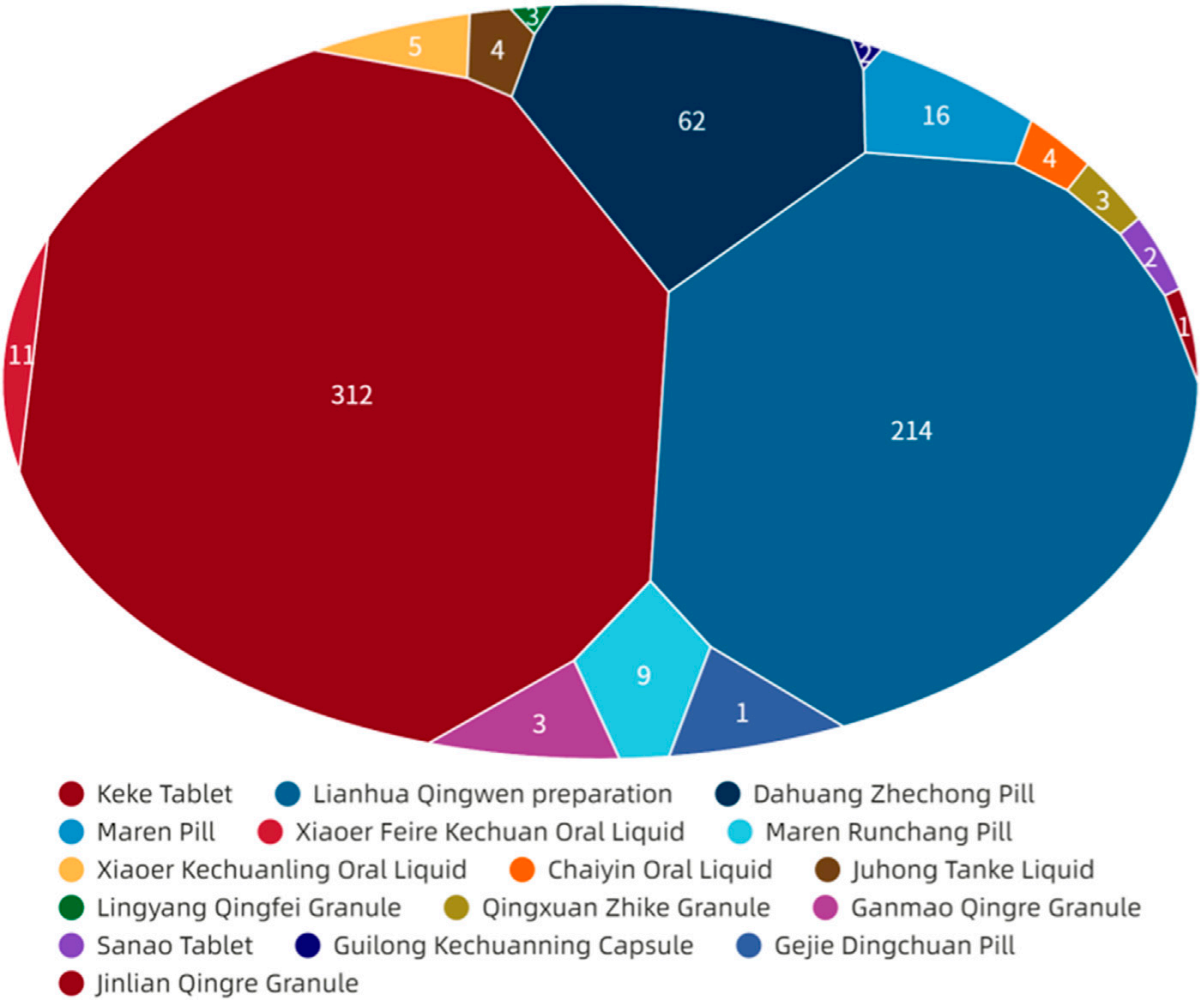
The quantity of ADEs of CPMs containing hydrogen cyanide.

**FIGURE 7:**
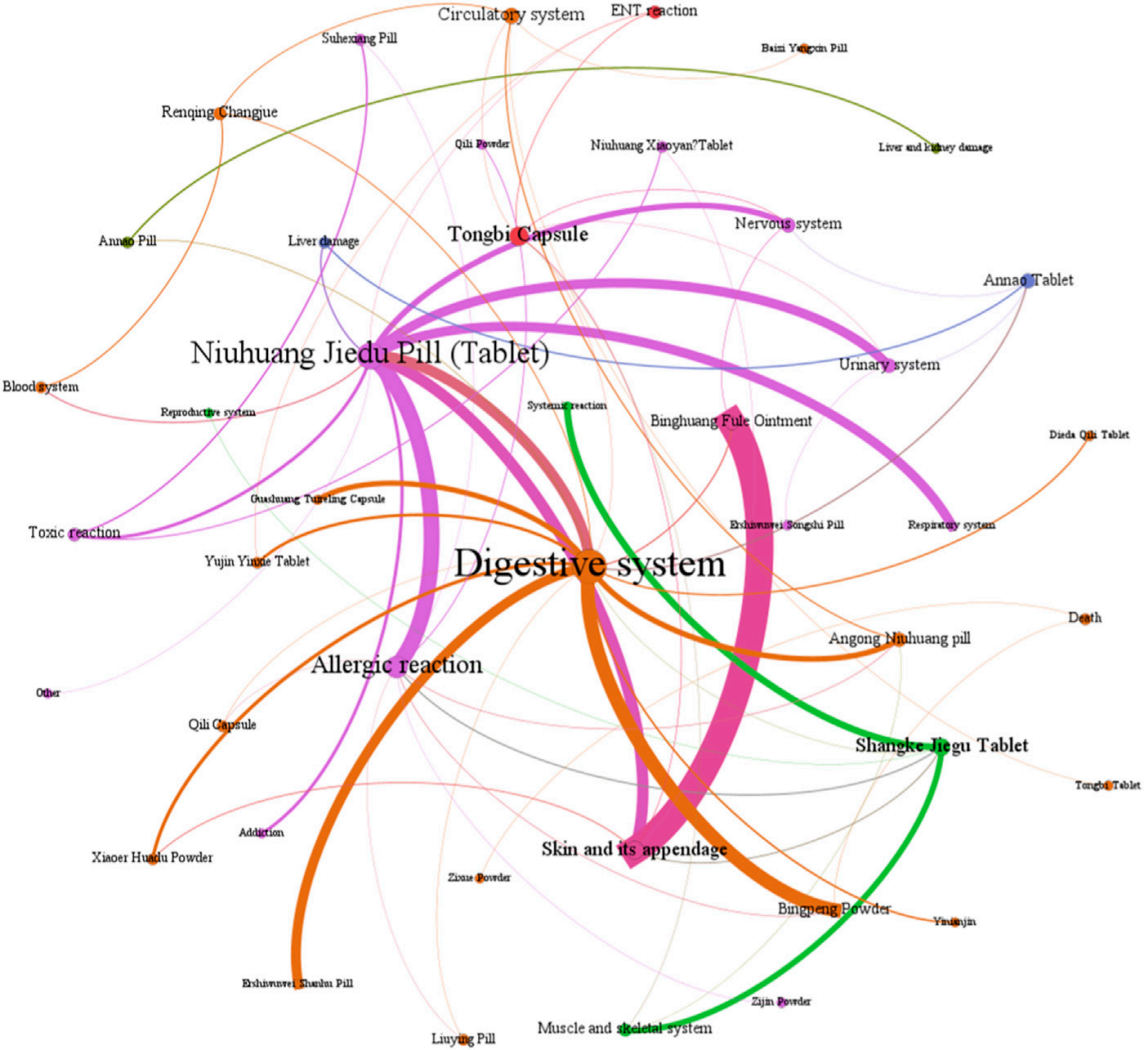
The network relationship between Chinese patent medicine containing mineral composition and related ADEs.

Figure 6 showed that digestive system symptoms were the most common ADEs among CPMs containing hydrogen cyanide, with a total of 337 cases (50.8%). This was followed by skin and its appendages (223 cases, 33.6%), and the nervous system (51cases, 7.7%) (see Figure 6; Supplementary Table S5). In addition, Figure 7 shows that Keke Table (312 cases), Lianhua Qingwen preparation (214 cases), and Dahuang Zhechong pill (62 cases) were the three varieties that contributed the most to the number of ADEs.

The analysis of the reasons for the ADEs found that the digestive symptoms were mainly due to the prescription’s properties, the patient’s weakened spleen and stomach, and inappropriate usage. Lianhua Qingwen preparation (tablet, granule), Keke tablet, and other heat-clearing medicines contain many cold and cool herbs that can easily damage the spleen and stomach (Xu et al., 2018; Hu et al., 2022). The Dahuang Zhechong pill, Maren Runchang pill, and Maren pill formulas contain laxative herbs, such as Rheum officinale Baill [Polygonaceae; Rhei Radix Et Rhizoma; Dahuang], Cannabis sativa L [Cannabaceae; Cannabis Fructus; Huomaren], Prunus armeniaca L [Rosaceae; Armeniacae Semen Amarum; Kuxingren]. These herbs may cause abdominal pain, increased stool frequency, loose stool, diarrhea, and other related symptoms. If the patient has a weak spleen or stomach, or engages in behaviors such as taking medicine on an empty stomach, taking medicine before meals, or taking multiple medicines, they may be at an increased risk of experiencing discomfort in their digestive system.

Skin and its appendages symptoms are primarily related to the patient’s allergic constitution and the prescribed medication. Patients with allergies or a history of allergies may be more susceptible to medication. Studies suggest that Ephedrae Herba and animal herbs may be the main allergens (Yang et al., 2015; Yi et al., 2018). CPMs such as Keke tablet containing *Ephedra sinica Stapf* [Ephedraceae; *Ephedrae Herba*; Mahuang], Dahuang Zhechong pill containing Ground Beetle [Corydiidae; *Eupolyphaga Steleophaga*; Tubiechong], Leech [Hirudinidae; *Hirudo*; shuizhi], and other animal herbs, have been found to cause a high proportion of skin symptoms in ADEs.

Additionally, studies have suggested a potential correlation between neurological symptoms and the use of Chinese Ephedrs Herb (Jamal et al., 2013). Ephedrine, a metabolite of *Ephedra sinica Stapf* can cause symptoms of central nervous and sympathetic excitation, including dizziness, nervousness, insomnia, palpitations, and increased blood pressure. ADEs associated with Keke tablet, Lianhua Qingwen preparations, and Sanao tablet, all of which contain *Ephedra sinica Stapf*, have shown specific nervous symptoms.

(3) Analysis of ADEs related to CPMs containing mineral composition

**FIGURE 8:**
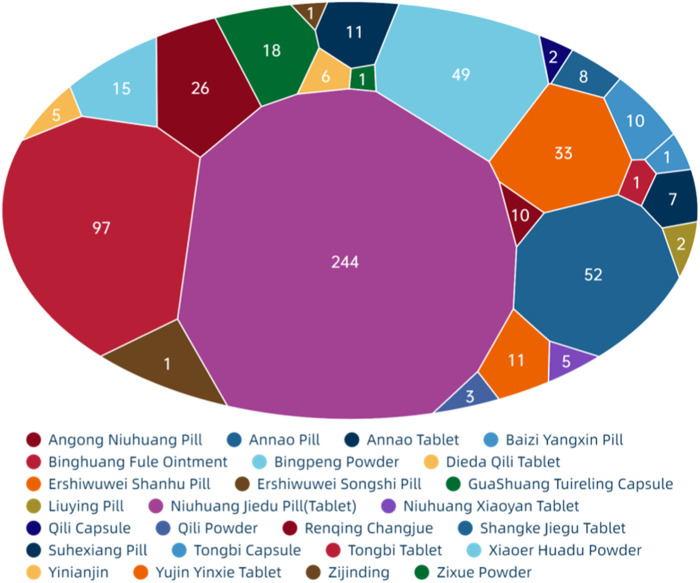
The quantity of ADEs of Chinese patent medicine containing mineral composition.

Figure 8 show that among the ADEs of CPMs containing mineral composition, the main types were symptoms of the digestive system, skin and its appendages, with 202 cases (32.6%) and 138 cases (22.3%), respectively. Allergic reactions accounted for 68 cases (11.0%) (Supplementary Table S6; Figure 8). The two varieties with the highest number of ADEs were Niuhuang Jiedu pill (tablet) (244 cases) and Binghuangfule ointment (97 cases) (see Figure 8). The Shangke Jiegu tablet and Bingpeng powder had a similar number of ADEs, with 52 and 49 cases respectively. In addition, two severe ADEs resulting in death were reported in CPMs containing mineral composition, including 1 case of Bingpeng powder and 1 case of Zixue powder.

The study identified a link between digestive symptoms and factors such as medication type, dosing method, and patient demographics. The primary function of this type of Chinese patent medicine is to clear heat and relieve spasms. The formula contains not only Cinnabar [Sulfides; *Cinnabaris*; Zhusha] and Red orpiment [Sulfides; *Realgar*; Xionghuang] but also other cold herbs. For example, the Ershiwuwei Shanhu pill includes Coral [Corallium; *Acropora echinate*; Shanhu], Pearl [Pteria; *Margarita*; Zhenzhu], Lazurite [*Lapis lazuli*; Qingjinshi], Nacre [Hyriopsis; *Margaritifera Concha*; Zhenzhumu], Fossil fragments [*Os Draconis*; Longgu], Smithsonite Calamine [Carbonates; *Calamina*; Luganshi], *Encephalolith* [Sciaenidae; Naoshi], and Magnetite [*Magnetitum*; Cishi]. Similarly, Bingpeng powder comprises Borneolum [*Borneolum Syntheticum*; bingpian], Borax [Boron; Pengsha], and Mirabilite [*Natrii Sulfas*; Mangxiao]. Guashuang Tuireling capsule contains Watermelon Frost [Cucurbitaceae; *Mirabilitum Praeparatum*; Xiguashuang], North Calcitum [Sulfates; *Gypsum Rubrum*; Hanshuishi], Gypsum [Sulfates; *Gypsum Fibrosum*; Shigao], Talc [Silicate; *Talcum*; Huashi], Magnetite [*Magnetitum*; Cishi], *Scrophularia ningpoensis* Hemsl [Scrophulariaceae; *Picriae Herba*; Kuxuanshen], Buffalo Horn [Bovidae; *Bubali Cornu*; shuiniujiao], Antelope Hom [Bovidae; *Saigae Tataricae Cornu*; lingyangjiao], and Borneolum [*Borneolum Syntheticum*; bingpian]. Angong Niuhuang pill contains Bezoar [Bovidae; *Bovis Calculus*; Niuhuang], Buffalo Horn [Bovidae; *Bubali Cornu*; shuiniujiao], Pearl [Pteria; *Margarita*; Zhenzhu], *Coptis chinensis* Franch [Ranunculaceae; *Picrorhizae Rhizoma*; Huhuanglian], *Scutellaria baicalensis* Georgi [Lamiaceae; *Scutellariae Radix*; Huangqin], *Gardenia jasminoides* J.Ellis [Rubiaceae; *Gardeniae Fructus*; Zhizi], *Curcuma aromatica* Salisb [Zingiberaceae; *Curcumae Radix*; Yujin], and Borneolum [*Borneolum Syntheticum*; bingpian]. Yujin Yinxie tablet contains *Curcuma aromatica* Salisb [Zingiberaceae; *Curcumae Radix*; Yujin], Sodium Sulfate Power [*Natrii Sulfas Exsiccatus*; Xuanmingfen], and *Strobilanthes cusia* (Nees) Kuntze [Acanthaceae; *Indigo Naturalis*; Qingdai]. The pungent or bitter smell of medicines can also lead to nausea or vomiting in patients (Chen and Wu, 2001; Zhou, 2009). Individuals with a weak spleen or stomach, particularly children, may experience digestive discomfort when consuming CPMs (Wang et al., 2016).

Skin and its appendage symptoms are primarily caused by Niuhuang Jiedu tablet/pill and Binghuang Fule ointment. The skin damage caused by Niuhuang Jiedu tablet/pill is due to allergic and toxic reactions. The use of Niuhuang jiedu tablet/pill has severe irrational medication phenomena such as unauthorized use, overdose, and long-term use, can lead to chronic arsenic poisoning, dermatitis rash, and other reactions (Tong et al., 2010). Arsenic poisoning can cause dry skin, rough, keratosis, papules, herpes, dermatitis, and pigmentation (Vergara-Gerónimo et al., 2021; Patel et al., 2021). The ADEs of Binghuang Fule ointment primarily include burning, itching, irritation, erythema, and desquamation, which can be alleviated through massage, saline compresses, or dosage reduction (Yang et al., 2022). The external dosage form of Binghuang Fule ointment is speculated to be the primary cause of ADEs. As the indications of the ointment are skin diseases like eczema, there may be confusion between disease complications and ADE symptoms during clinical observation.

Allergic reactions are primarily associated with the formula, administration, and dosage form. Some studies suggest that anaphylactic shock may be related to realgar. Improper processing, preparation, or storage can cause the arsenic in realgar to act on proteins and cause allergies or even shock (Li and Liu, 2018). The starfish found in the ingredients of Shangke Jiegu tablet contains various proteins and polypeptides, which have the potential to cause allergic reactions (Ihama et al., 2014). Topical medicines, such as Bingpeng powder and Qili powder, can cause allergic contact dermatitis. Patients with allergic histories, as well as children and the elderly, are particularly sensitive to these medicines.

Additionally, this study analysed the causes of two ADE-related deaths (Yang, 1991; Ma and Jin, 1994). In the case of Bingpeng powder, a newborn developed systemic rashes, positive fecal occult blood, vomited foam, hypothermia, confusion, cold and wet skin, cyanosis of the extremities, ecchymosis of the buttocks and thighs, and other toxic manifestations after ten consecutive days of external use. In the case of Bingpeng powder, a 17-day-old newborn suffered from convulsions, cyanosis, rales indicative of phlegm in the lungs, and significant abdominal distension following three consecutive instances of overdosing on Zixue powder. This highlights the importance of rational medication, especially for special populations such as newborns.

(4) Analysis of ADEs related to CPMs containing Araceae metabolites

**FIGURE 9:**
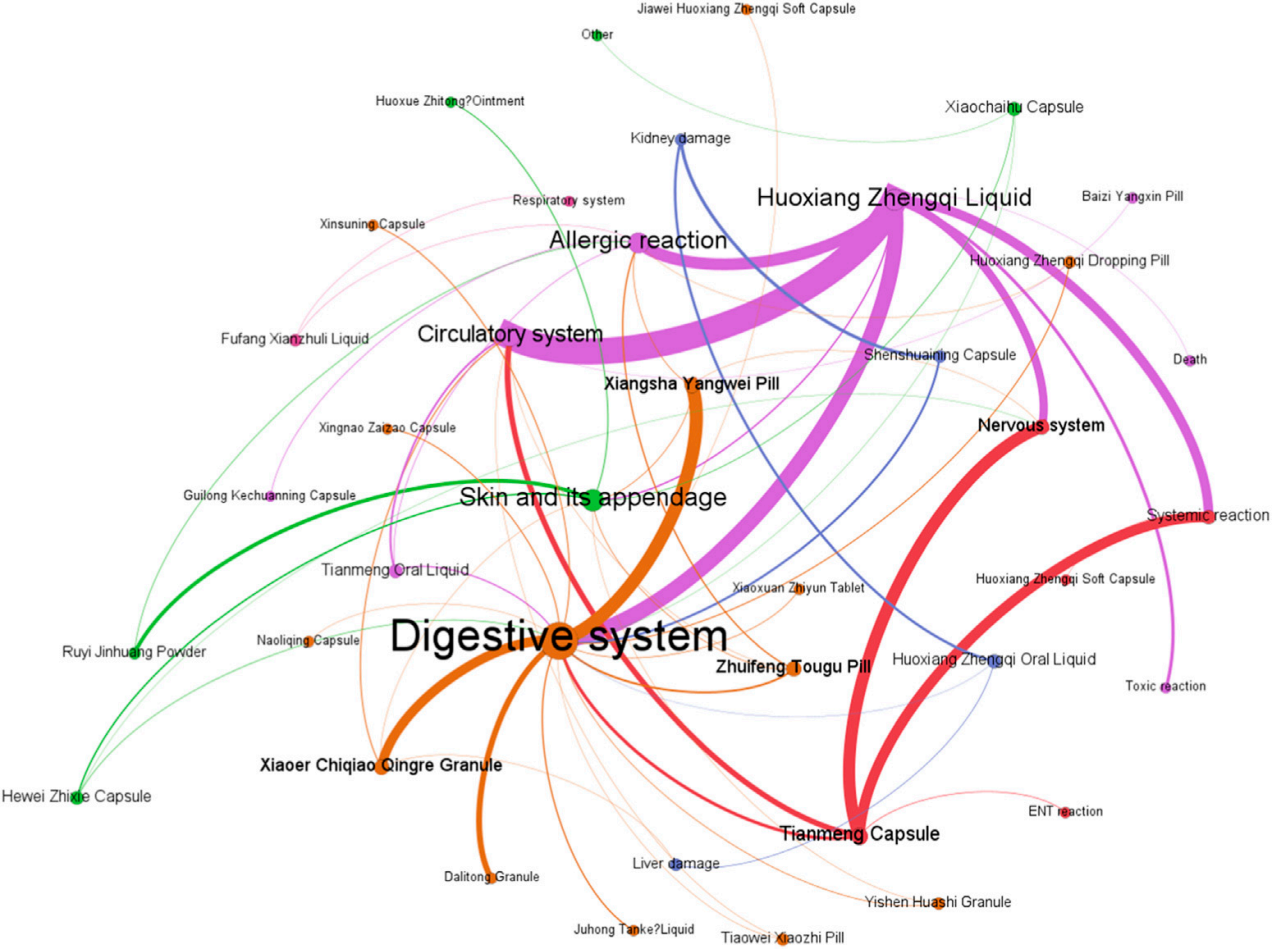
The network relationship between CPMs containing Araceae metabolites and related ADEs.

**FIGURE 10:**
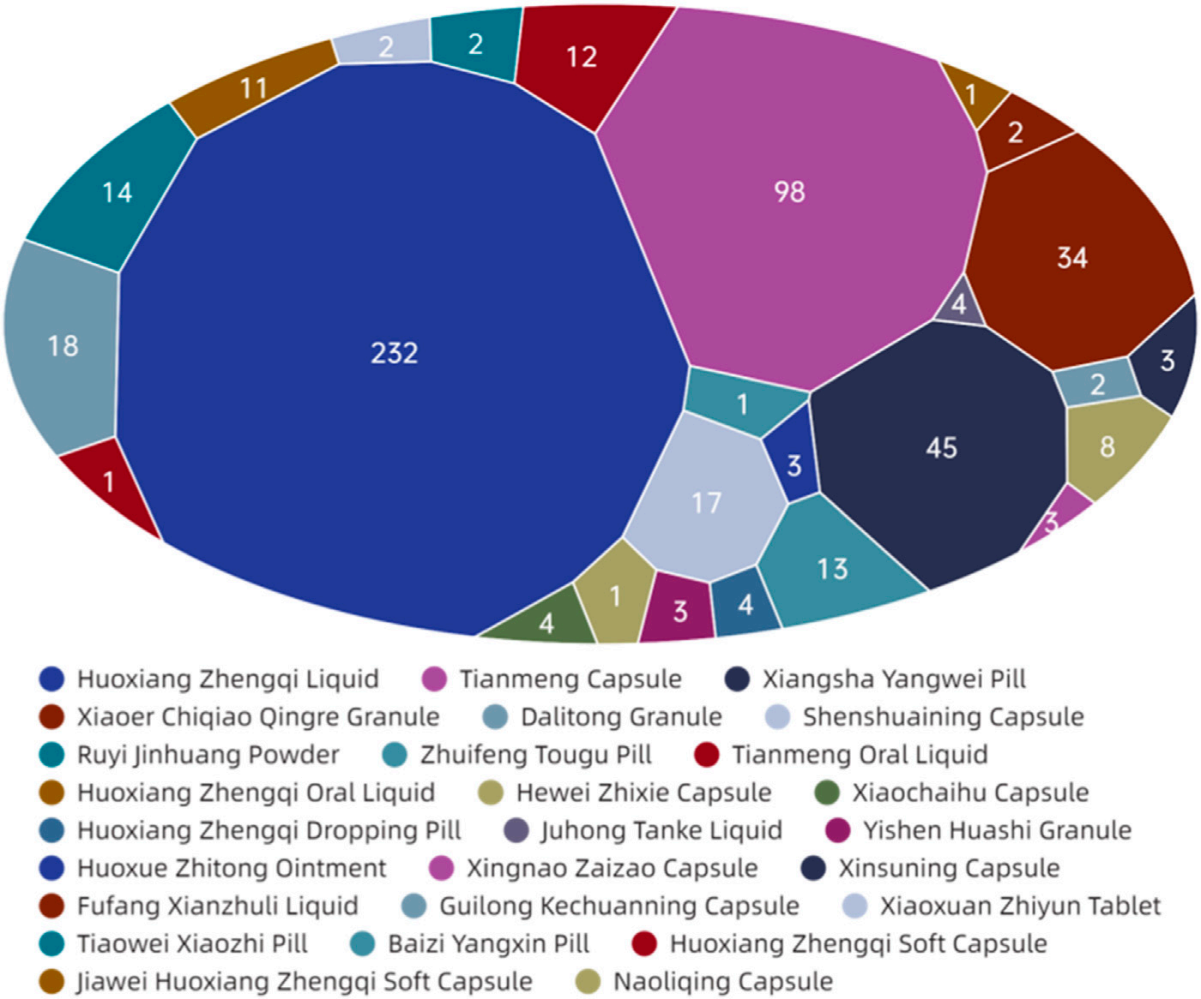
The quantity of ADEs of Chinese patent medicine containing Araceae metabolites.

Figure 9 demonstrates that among the ADEs associated with CPMs containing Araceae metabolites, the primary symptom types relate to the digestive and circulatory systems, accounting for 185 cases (34.4%) and 108 cases (20.1%) respectively. Additionally, the nervous system (64 cases, 11.9%) and systemic reactions (62 cases, 11.5%) are also impacted (see Supplementary Table S7; Figure 9). Huoxiang Zhengqi liquid (232 cases), Tianmeng capsule (97 cases), and Xiangsha Yangwei pill (45 cases) are the most frequently implicated in ADEs (see Figure 10). Furthermore, a fatal ADE has been reported in association with the use of Huoxiang zhengqi liquid, a specific type of Chinese patent medicine.

ADEs related to the digestive system are primarily attributed to specific medicinal ingredients or excipients present in prescription. Certain patients may encounter a bitter aftertaste following the administration of Huoxiang Zhengqi liquid, attributable to its palatability. Additionally, the excipients within this liquid include ethanol, which has the potential to induce digestive damage if consumed in excessive amounts or by specific populations, such as those with pre-existing liver conditions or alcohol intolerance. Studies have indicated that *Wurfbainia villosa* (Lour.) Škorničk. and A.D.Poulsen [Zingiberaceae; *Amomi Fructus*; Sharen], Tangerine Peel [Rutaceae; *Citri Reticulatae Pericarpium*; Chenpi], *Atractylodes lancea* (Thunb.) DC. [Asteraceae; *Atractlodis Rhizoma*; Cangzhu), and *Pogostemon cablin* (Blanco) Benth. [Asteraceae; *Pogostemonis Herba*; Guanghuoxiang] promote the secretion of gastric digestive juice, enhance digestive power, and may increase stool frequency (Zhang et al., 2008). The herbs such as Tangerine Peel [Rutaceae; *Citri Reticulatae Pericarpium*; Chenpi], *Dolomiaea costus* (Falc.) Kasana & A.K.Pandey [Asteraceae; *Aucklandiae Radix*; Muxiang], *Citrus aurantium* L [Rutaceae; *Aurantii Fructus Immaturus*; Zhishi], *Wurfbainia villosa* (Lour.) Škorničk. and A.D.Poulsen [Zingiberaceae; *Amomi Fructus*; Sharen] in Xiangsha Yangwei pill can excite the gastrointestinal smooth muscle, enhancing contraction force and accelerating peristalsis. Furthermore, herbs such as *Wurfbainia villosa* (Lour.) Škorničk. and A.D.Poulsen [Zingiberaceae; *Amomi Fructus*; Sharen], Tangerine Peel [Rutaceae; *Citri Reticulatae Pericarpium*; Chenpi], *Atractylodes macrocephala* Koidz. [Asteraceae; *Atractylodis Macrocephalae Rhizoma*; Baizhu], *Pogostemon cablin* (Blanco) Benth. [Asteraceae; *Pogostemonis Herba*; Guanghuoxiang] have been found to promote gastric secretion, which may result in an increase in stool frequency.

The Tianmeng capsule primarily induces symptoms in the nervous system and systemic reactions. These symptoms include insomnia, dizziness, lethargy, emotional depression, and fatigue. It is possible that complications ADEs may be mistaken for one another during clinical observation due to the neurological indications of the Tianmeng capsule.

Huoxiang zhengqi liquid can cause symptoms in the circulatory system and systemic reaction. It is crucial to highlight that the excipients present in Huoxiang Zhengqi liquid comprise ethanol, a constituent that may precipitate ethanol poisoning in cases of misuse or if the patient exhibits intolerance or allergy towards ethanol. In addition, combining Huoxiang zhengqi capsule with certain antibiotics, such as tinidazole, metronidazole, furazolidone, ceftriaxone sodium, and cefoperazone sodium, can cause a disulfiram-like reaction (intoxication reaction) (Ren et al., 2014). Finally, the analysis of the cause one ADE-related death was finally conducted (Yang and Deng, 1997). The instructions for Huoxiang Zhengqi liquid indicated ‘oral administration’, however, the method of medication in the fatal case was intravenous injection. This highlights the potential for serious adverse events resulting from incorrect administration routes.

(5) Analysis of ADEs related to CPMs containing diester aconitine metabolites

**FIGURE 11:**
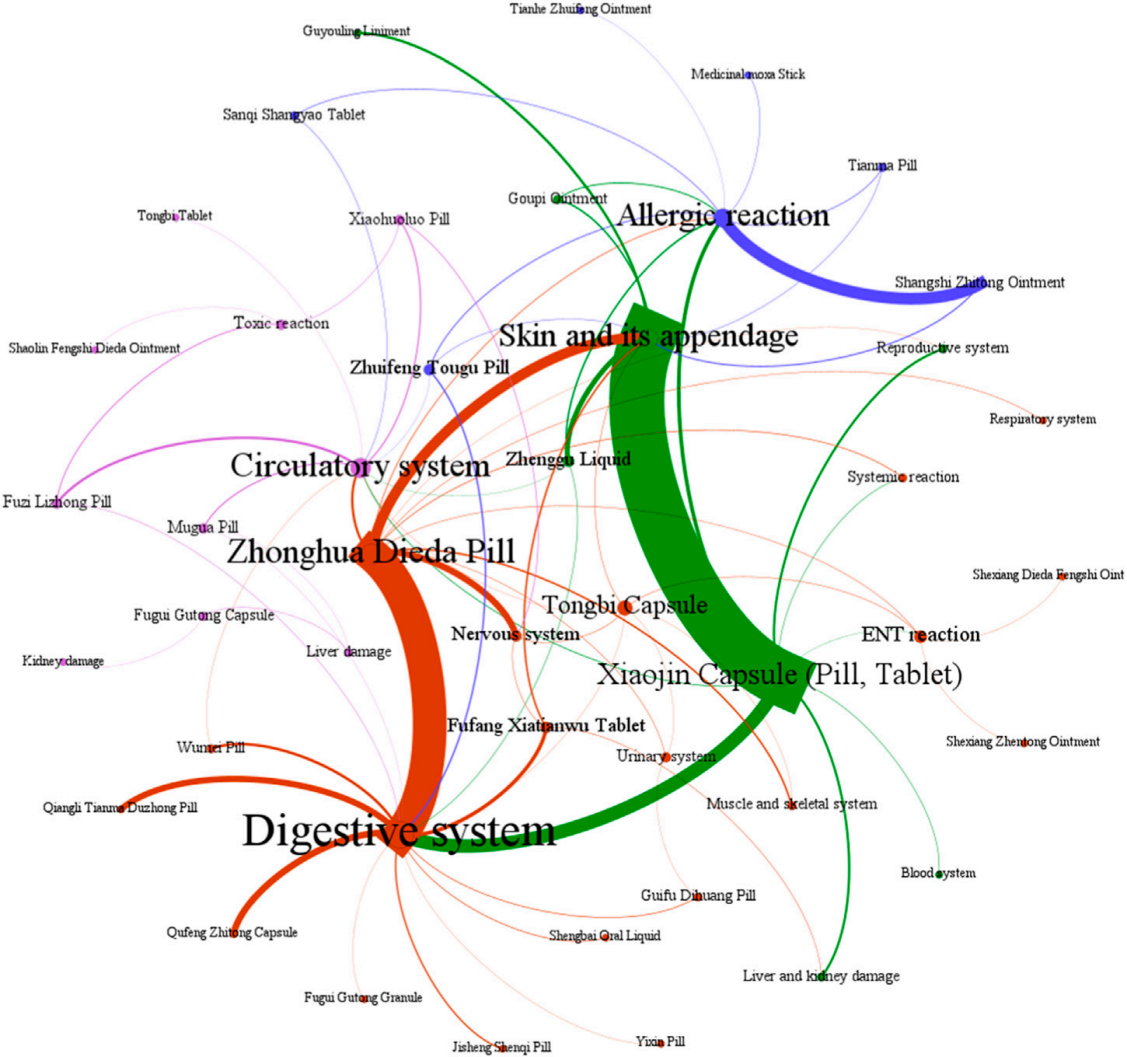
The network relationship between CPMs containing diester aconitine metabolites and related ADEs.

**FIGURE 12:**
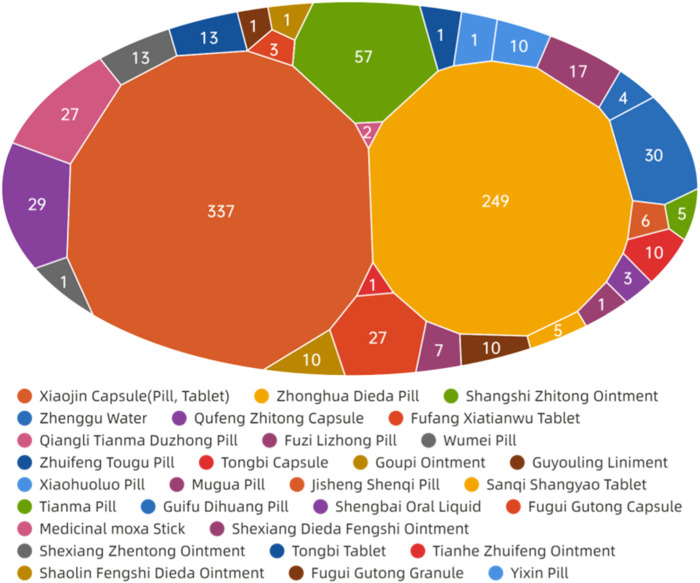
The quantity of ADEs of CPMs containing diester aconitine metabolites.

The data presented in Figure 11 demonstrate that ADEs associated with CPMs containing diester aconitine metabolites primarily impact the digestive system (314 cases, 35.6%), the skin and its appendages (338, 38.4%), and also induce allergic reactions (93 cases, 10.6%). Additionally, the circulatory system (41 cases, 4.7%) and nervous system (35 cases, 4.0%) among the affected areas (Supplementary Table S8; Figure 11, Figure 12). Notably, Xiaojin capsule (pill, tablet) (337 cases) and Zhonghua Dieda pill (249 cases) were the two most prevalent types of medicines contributing to these ADEs.

The literature of Chinese patent medicine containing diester aconitine metabolites rarely analyses the reasons for the digestive system symptoms associated with ADEs. The ADEs of the Zhonghua Dieda pill mainly cause gastrointestinal damage, which is consistent with previous literature reports (Luo et al., 2018). It is recommended that manufacturers make timely additions to the adverse reaction section of the instructions. To ensure safe medication, physicians and pharmacists must carefully read the instructions and strictly control the indications, dosage, and course of treatment. Dosage should be reduced for patients with physical deficiencies and gastrointestinal dysfunction. It is also important to educate patients to guarantee the security of clinical medication.

The prescription of these medicines and their dosage forms are primarily responsible for the ADEs on the skin and its appendages, as well as triggering allergic reactions. Studies have indicated that certain topical formulations, such as Shangshi Zhitong ointment, Zhenggu water, Goupi ointment, Guyouling liniment, and Moxa stick, have the potential to elicit skin allergic reactions. Xiaojin preparation (tablet, capsule, pill) contains abundant macromolecular substances, such as animal and plant proteins and polypeptides, which can easily trigger allergic reactions. It is acknowledged that the toxic alkaloids inherent in Momordicae Semen and Aconiti Kusnezoffii Radix Cocta have the potential to induce ADEs when these herbs are not subjected to proper processing. Furthermore, Olibanum, Myrrha, and Liquidambar balsam contain resinous substances that act as small molecule haptens, which can provoke telangiectasia and heightened permeability of the skin, ultimately resulting in the manifestation of skin rash, dermatitis, or angioedema (Al-Suwaidan et al., 1998; Greve et al., 2016). Besides, studies have shown that Xiaojin pills may have a higher incidence of ADEs compared to other dosage forms. This suggests that pills carry a greater risk of ADEs. Furthermore, the highest proportion of ADRs for Xiaojin preparations has been found in patients aged 60 and above, indicating that elderly patients with poor physical fitness are at an increased risk of adverse reactions (Xin, 2017).

The symptoms affecting the circulatory and nervous systems are primarily caused by the diester aconitine metabolites. These metabolites can cause toxic effects such as numbness of the mouth and tongue, tachycardia, and facial edema. When aconite is decocted for a long period of time, the diester aconitine metabolites present in it are hydrolyzed into aconine. The cardiotonic metabolites are not destroyed, but the toxicity is significantly reduced, resulting in a clear cardiotonic effect. Previous studies have shown that Fuzi Lizhong pill can cause the following poisoning symptoms (Karturi et al., 2016; Coulson et al., 2017; Jeon et al., 2020): ① numbness of the lips and limbs may occur after paralysis of peripheral nerves; ② nausea, vomiting, salivation, sweating, diarrhea, and other symptoms of vagus nerve excitation may occur; ③ various arrhythmias caused by excitation of the vagus nerve and frequent ectopic rhythms caused by direct toxic effects on the myocardium. Diester aconitine metabolite poisoning can occur in certain circumstances, such as in sensitive individuals, due to improper processing of herbs, or as a result of an overdose of medication.

## 5 Discussion

Since the emergence of COVID-19, the trade of traditional Chinese medicine products has quickly resumed stable development after overcoming severe market changes. According to a retrospective study by Zhang et al., CPMs have been effective in fighting COVID-19, with heat-clearing medicines being the most commonly used (Zhang et al., 2020). CPMs that contain toxic ingredients are essential in treating acute critical illness with TCM or integrative medicine. The therapeutic thought of “fighting poison with poison” is an important part of TCM theory. CPMs containing toxic ingredients have played an indispensable role in the clinical treatment of TCM, especially in the treatment of difficult and complex diseases. For example, arsenic trioxide (As2O3), the main effective component of Arsenic, has a significant inhibitory effect on leukemia cells. It can be seen that the toxicity and effectiveness of CPMs containing toxic components coexist, and the existence of toxic components should not hinder their clinical use. This study holds that toxic ingredients, toxic Chinese medicines and toxic CPMs are not equivalent.

The toxicity ranking method used in TCM is based on practical experience recorded in ancient Materia Medica. TCM marks the toxicity of its medicines as ‘strong toxicity’, ‘toxic’, and ‘small toxicity’, which reflects the inherent toxicity of Chinese patent medicine containing toxic ingredients. Currently, research on Chinese patent medicine containing toxic ingredients mainly focuses on statistical analysis of internal medication in hospitals. However, it is important to note that these studies may be influenced by factors such as hospital specialty characteristics and drug catalogs, which may not provide a complete representation of the overall clinical risk associated with CPMs containing toxic ingredients. Therefore, this paper intends to directly observe the overall risk situation presented in the clinical use of Chinese patent medicine containing toxic ingredients, objectively analyze the factors and causes of risk, and provide reference for drug supervision.

This study identified four toxic ingredients commonly found in Chinese Pharmacopoeia and sorted out the corresponding toxic herbs and CPMs. The diester aconitine metabolites, hydrogen cyanide, and mineral composition were found to possess both toxicity and efficacy. Processing, compounding, and rational medication can enhance the effects and reduce the toxicity of these ingredients. Furthermore, as Paracelsus, the founder of modern toxicology, said, “Solely the dose determines that a thing is not a poison”. The appropriate clinical utilization can effectively mitigate toxicity and optimize therapeutic efficacy. However, the relationship between the dose and safety of toxic ingredients and toxic medicinal materials is not equivalent to that of Chinese patent medicine containing toxic ingredients due to factors such as processing and concerted application. Currently, there remains significant room for improvement in the basic research on acute and chronic toxicity of CPMs when compared to the research status of toxic ingredients and toxic medicinal materials, which is crucial for ensuring the clinical safety of CPMs containing toxic ingredients.

**FIGURE 13:**
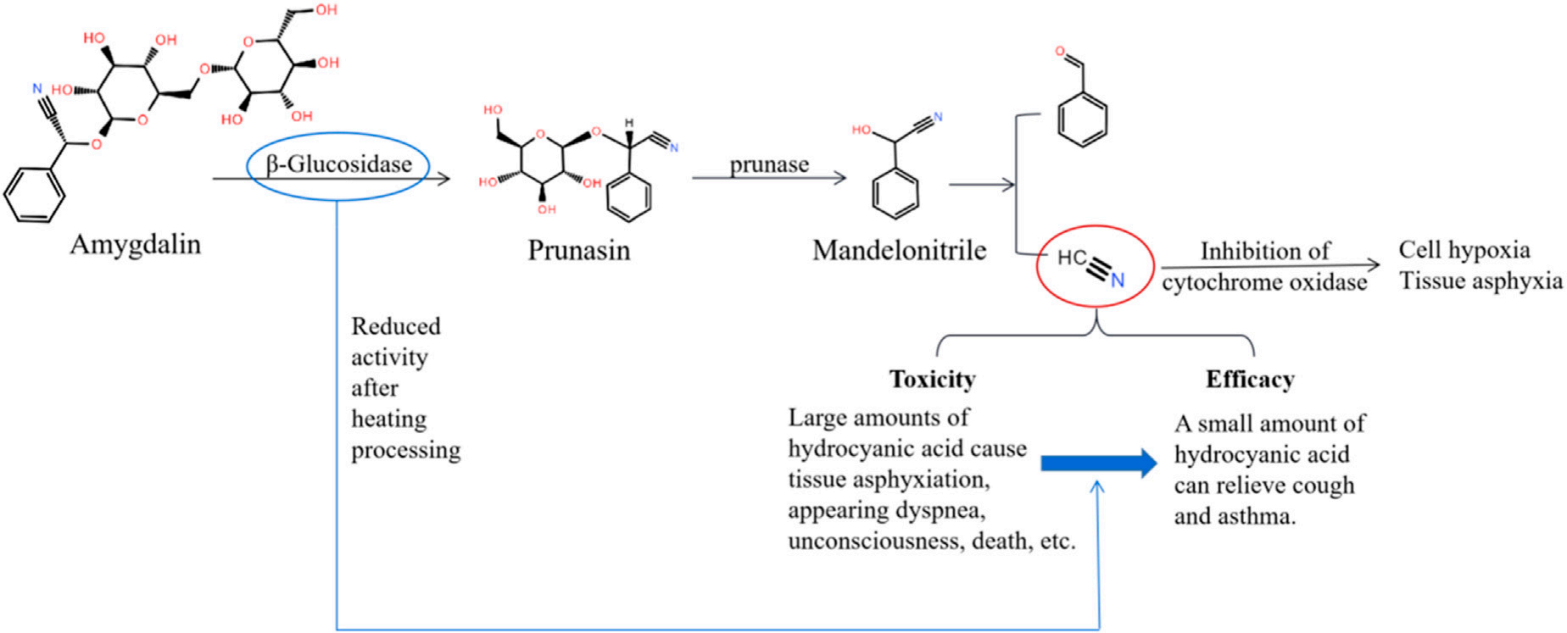
The metabolism of amygdalin, as well as the toxicity and efficacy of hydrogen cyanide.

Hydrogen cyanide is produced during the metabolism of amygdalin in herbs such as *Prunus armeniaca* L [Rosaceae; *Armeniacae Semen Amarum*; Kuxingren]. While a small amount of hydrogen cyanide can relieve cough and asthma, large amounts can affect cellular respiration and lead to tissue hypoxia (Mani et al., 2019; Figure 13).

**FIGURE 14:**
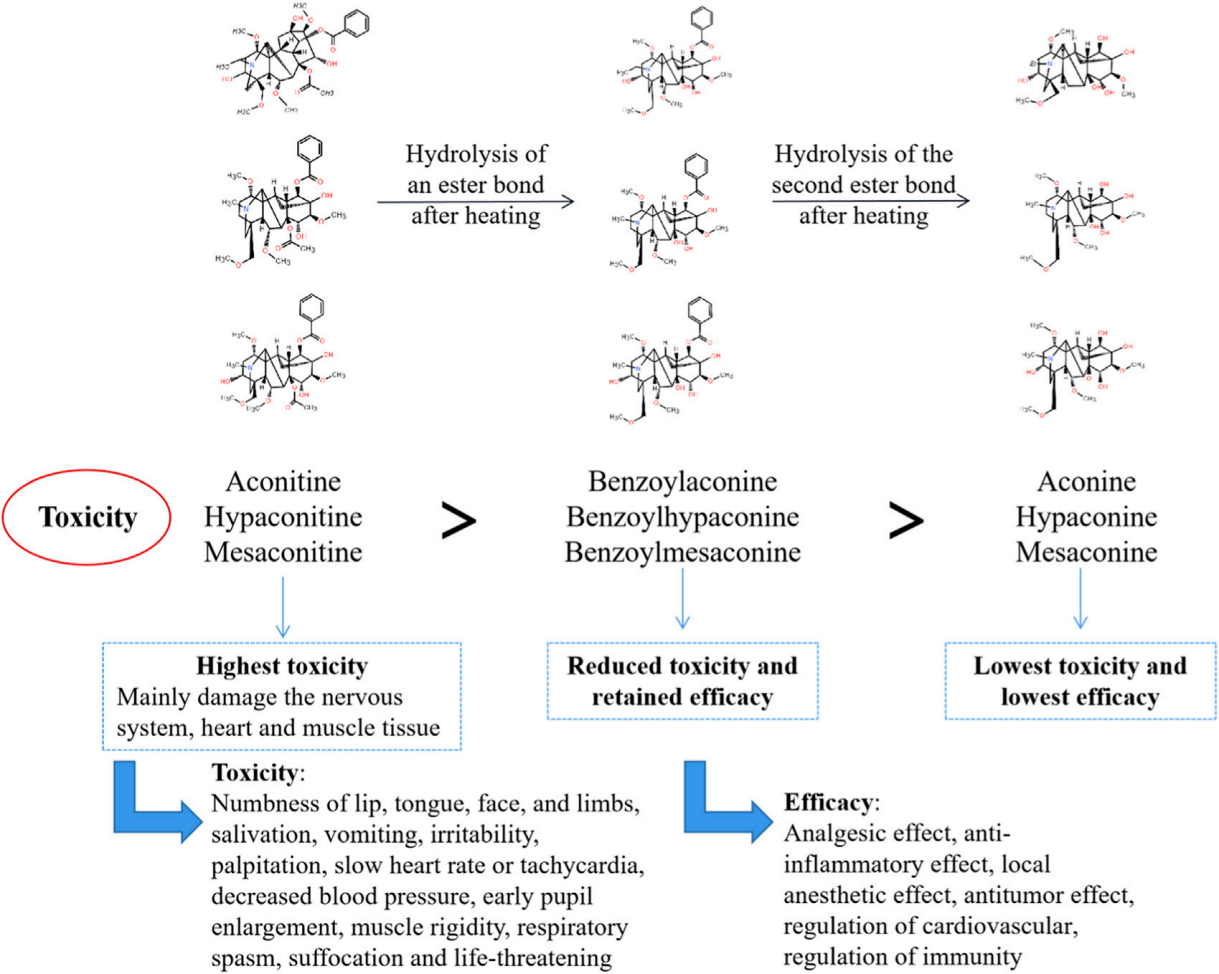
The metabolism of aconitine metabolites and toxicity-efficacy relationship.

The diester aconitine metabolites, namely aconitine, mesaconitine, and hypaconitine, possess significant cardiotoxic and neurotoxic properties. These diester aconitine metabolites, namely aconitine, mesaconitine, and hypaconitine, can be converted into monoester aconitine metabolites, specifically benzoylaconine, benzoylmesaconine, and benzoylhypaconine, through hydrolysis. The toxicity of these monoester aconitine metabolites is reduced to 1/200 of the diester aconitine metabolites. In addition, hydrolysis can convert the monoester aconitine metabolites to alkamine metabolites, namely aconine, mesaconine, and hypaconine. These metabolites are less toxic, with a toxicity level of 1/2,000 compared to the diester aconitine metabolites (Li et al., 2022) (see Figure 14).

**FIGURE 15:**
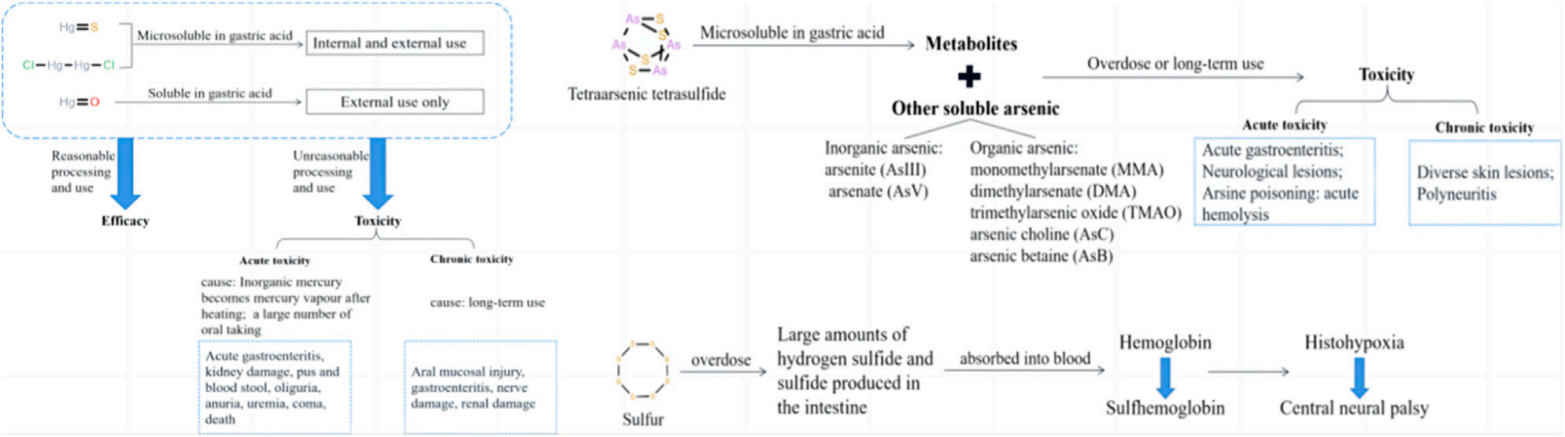
The metabolism of mineral composition and toxicity-efficacy relationship.

The mineral composition of the herbs include mercury, arsenic, and sulfur. Mercuric sulfide and dimercury dichloride exhibit slight solubility in an acidic environment, and while they may have certain applications, their use, both internally and externally, is highly regulated due to potential toxicity. In contrast, mercuric oxide, which is soluble in acidic conditions, is strictly limited to external use only. It is crucial to note that improper preparation or prolonged exposure to any of these compounds can result in acute or chronic toxicity, emphasizing the need for strict adherence to safety protocols and controlled conditions (Jain et al., 2019). Acute toxicity is characterised by severe gastroenteritis and kidney damage. Chronic toxicity typically arises from prolonged use of related medication, resulting in the accumulation of mercury over time. This is more common than acute toxicity (see Figure 15). Arsenic composition has low solubility in gastric acid. However, the dissolved portion along with other soluble forms of arsenic present in realgar can be absorbed into the body, potentially causing acute and chronic toxicity in cases of overdose or long-term exposure. Acute toxicity presents as severe gastroenteritis, neurological lesions, and arsine poisoning. Chronic toxicity mainly manifests as diverse skin lesions and polyneuritis due to chronic arsenic poisoning (Tian et al., 2019). Excessive sulfur entering the intestine is rapidly oxidized into non-toxic sulfide (sulfate or thiosulfate), which is excreted through the kidneys and intestines. Unoxidized hydrogen sulfide has toxic effects. Hydrogen sulfide can cause obstacles in the redox process of cells, leading to tissue hypoxia and central neural palsy (Dattilo et al., 2022).

**FIGURE 16:**
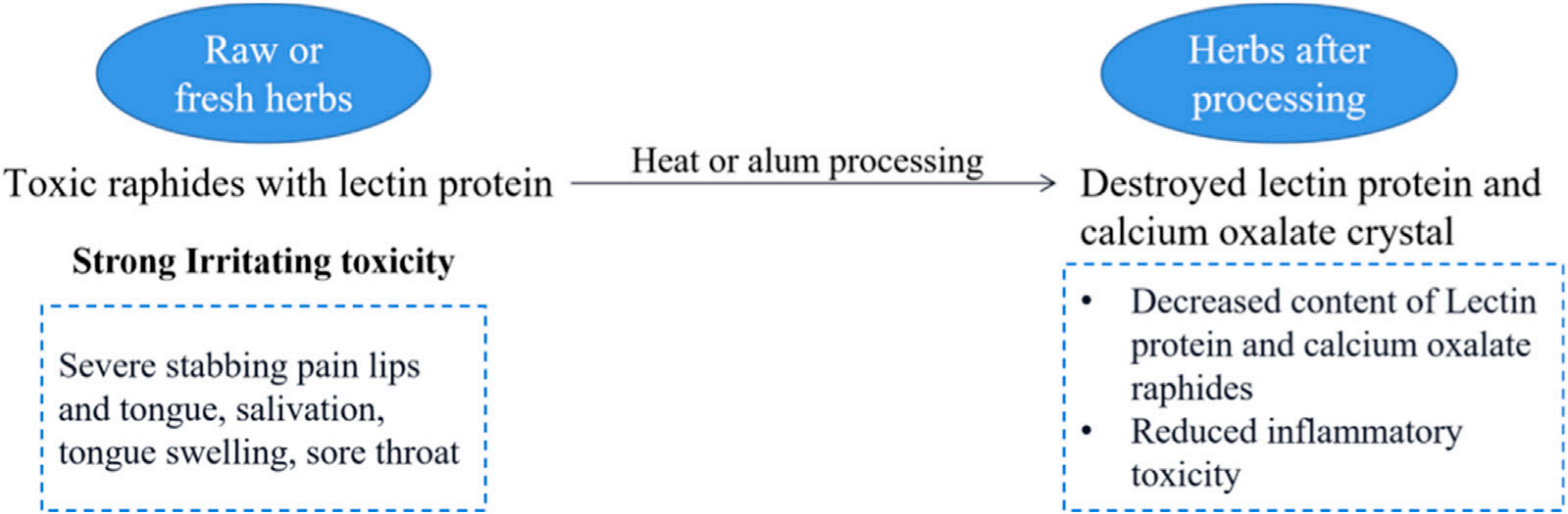
The processing attenuation mechanism of Araceae metabolites.

*Pinellia ternata* (Thunb.) Makino [Araceae; *Pinelliae Rhizoma*; Banxia], *Arisaema heterophyllum* Blume [Araceae; *Arisaematis Rhizoma Preparatum*; Zhitiannanxing], *Sauromatum giganteum* (Engl.) Cusimano & Hett. [Araceae; *Typhonii Rhizoma*; Baifuzi], and *Arisaema erubescens* (Wall.) Schott [Araceae; *Arisaematis Rhizoma*; Tiannanxing] are herbal medicines from the Araceae family. They have substantial irritant toxicity when consumed raw or fresh, so they are mainly used after processing for oral medication. Studies have revealed that these CHMs contain toxic raphides, which harbor lectin proteins and possess potent pro-inflammatory toxicity. However, alum or heating procedures can destroy the structure of these toxic raphides, significantly reducing their inflammatory toxicity (Su et al., 2021) (see Figure 16).

However, there are limitations to this research. Firstly, the original clinical studies did not include degree classification or cause analysis of ADEs, making it difficult to conduct a more in-depth statistical analysis. Four types of Chinese patent medicine containing toxic ingredients were selected as the research objects. CPMs containing animal metabolites were excluded from the study due to their small sample size. n addition to the four common CPMs examined in this study, there are other formulations that warrant caution during clinical use, including the CPMs containing *Crotonis fructus*, *Daturae flos*, *Euphorbiae semen*, *Hyoscyami semen*, *Kansui radix, etc.* Therefore, a deeper investigation into these types of CPMs is imperative. Besides, follow-up studies will focus on the association between dosage and safety of CPMs containing toxic ingredients to demonstrate the current status of dosage as a clinical risk factor. Finally, this study did not analyse the clinical benefits of CPMs containing toxic ingredients from a ‘risk-benefit’ perspective. The current analysis is yet to be comprehensive and lacks a strong guiding effect on the rational and safe clinical use of CPMs that contain toxic ingredients. In the future, our goal is to strengthen the risk-benefit analysis of CPMs containing toxic ingredients. We intend to delve into the intricate interplay between these toxic components, the associated risk factors, and their clinical benefits. This endeavor will furnish clinicians with high-quality evidence to guide the rational and safe use of these medicines in clinical practice.

## 6 Conclusion

**FIGURE 17:**
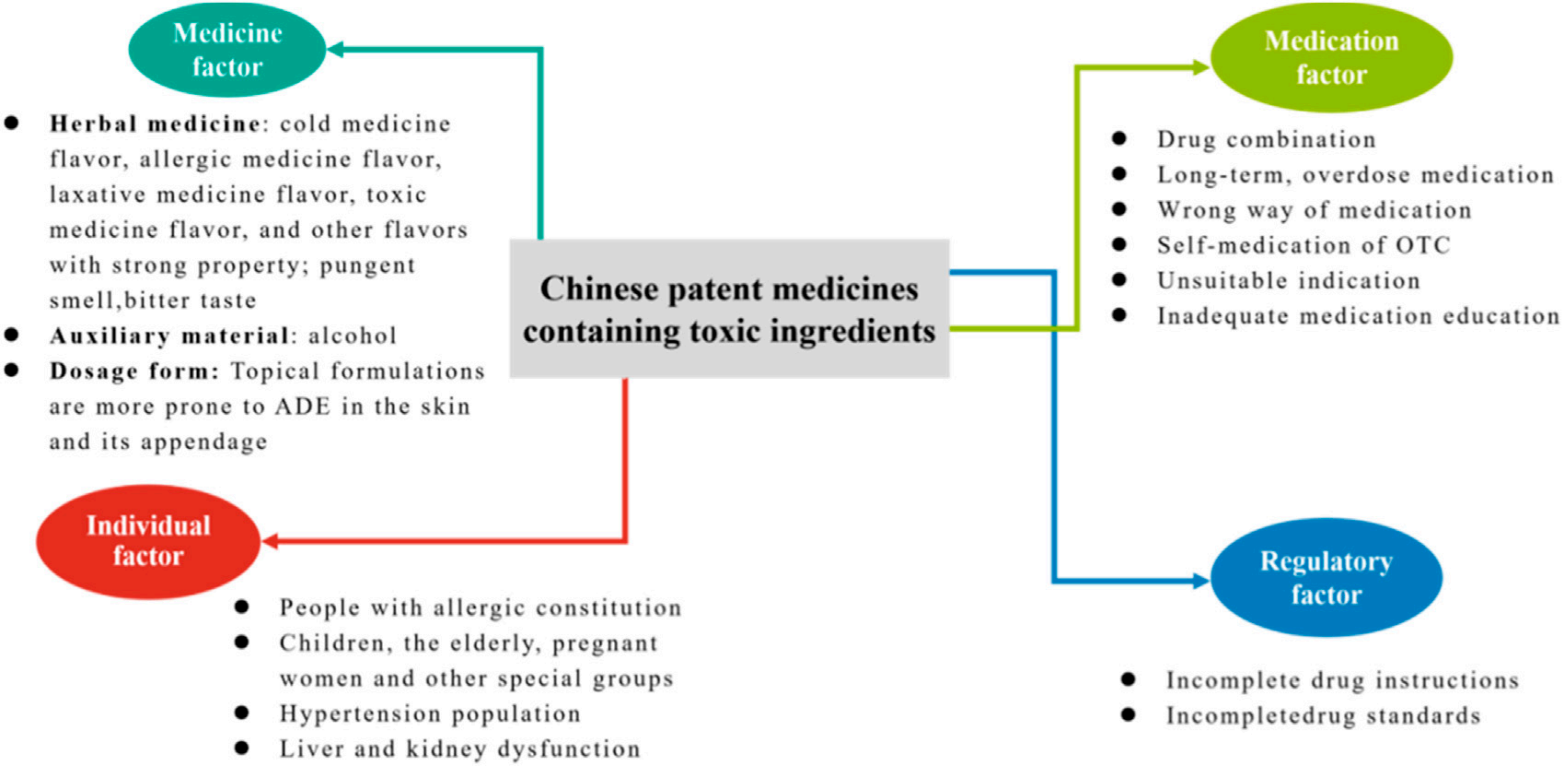
The clinical risk of CPMs containing toxic ingredients.

The purpose of this study was to evaluate the clinical safety profile of CPMs that contain toxic ingredients and to identify the risk characteristics of CPMs that contain different types of toxic ingredients. The study found that ADEs related to CPMs containing toxic ingredients were mainly associated with the digestive system, skin and its appendages, and allergic reactions. The study demonstrates that CPMs containing toxic ingredients pose clinical risks due to medicine, medication, individual, and regulatory factors (see Figure 17). In terms of the medicinal factor, the study revealed that digestive discomfort was the most prevalent adverse drug event (ADE), which was likely attributed to the potent therapeutic properties and inherent toxicity of the medications involved. This is due to the fact that these medicines come into direct contact with the stomach lining and undergo digestion following administration. In addition, CPMs containing diester aconitine or mineral compositions have been linked to ADEs, primarily due to the toxicity of their ingredients, including the risks of arsenism and aconitine poisoning. CPMs containing hydrogen cyanide and Araceae metabolites have been associated with fewer ADEs due to improved safety of the herbal pieces after processing. Medication factors are reflected in all types of CPMs. When administering CPMs containing toxic ingredients in conjunction with Western medicines or other CPMs of similar therapeutic effects, it is crucial to be mindful of potential ingredient repetition or interactions. Long-term or excessive medication can lead to poisoning from toxic ingredients. It is important to seek medical advice before taking any medication. For commonly used over-the-counter drugs, such as Huoxiang Zhengqi liquid and Bingpeng powder, patients are more likely to experience adverse drug events if they self-medicate.

## Data Availability

Publicly available datasets were analyzed in this study. This data can be found here: China National Knowledge Infrastructure (CNKI) and Wanfang Database.

## Abbreviations

ADEs, adverse events; ADRs, adverse reactions; TCM, traditional Chinese medicine; NCADRM, National Center for Adverse Drug Reaction Monitoring; CHMs, Chinese herbal medicines; CPMs, Chinese patent medicines; AA, aristolochic acid; CNKI, the China National Knowledge Infrastructure; ENT Reaction, Ear, Nose, and Throat Reaction.

## References

[B1] Al-Suwaidan S. N. Gad el Rab M. O. Al-Fakhiry S. Al Hoqail I. A. Al-Maziad A. Sherif A. B. (1998). Allergic contact dermatitis from myrrh, a topical herbal medicine used to promote healing. Contact Dermat. 39 (3), 137. 10.1111/j.1600-0536.1998.tb05867.x 9771992

[B2] Bao J. Xie Z. Liu S. Huang L. Sun J. Tang Y. (2021). Treatment effects of integrated TCM and western medicine treatment scheme on COVID-19: a single-armed clinical trial. Clin. Complement. Med. Pharmacol. 1 (1), 100009. 10.1016/J.CCMP.2021.100009 38620955 PMC8590636

[B3] Charen E. Harbord N. (2020). Toxicity of herbs, vitamins, and supplements. Adv. Chronic. Kidney. Dis. 27 (1), 67–71. 10.1053/j.ackd.2019.08.003 32147004

[B4] Chen W. Wu Y. (2001). Therapeutic effect of Guashuang Tuireling on Infantile fever. J. Jianghan Univ. (4), 21–22.

[B5] Coulson J. M. Caparrotta T. M. Thompson J. P. (2017). The management of ventricular dysrhythmia in aconite poisoning. Clin. Toxicol. (Phila) 55 (5), 313–321. 10.1080/15563650.2017.1291944 28421842

[B6] Dattilo M. Fontanarosa C. Spinelli M. Bini V. Amoresano A. (2022). Modulation of human hydrogen sulfide metabolism by micronutrients, preliminary data. Nutr. Metab. Insights. 15, 11786388211065372. 10.1177/11786388211065372 35023928 PMC8743967

[B7] Greve H. L. Kaiser M. Brun R. Schmidt T. J. (2016). Search for new antiprotozoal leads in oleo-gum-resins from plants of the family Burseraceae: indian frankincense and myrrh. Planta Med. 81 (S 01), s1–s381. 10.1055/s-0036-1596411

[B8] Hu C. He B. Gong F. Liang M. Zhao D. Zhang G. (2022). The adverse reactions of Lianhua Qingwen capsule/granule compared with conventional drug in clinical application: a Meta-analysis. Front. Pharmacol. 13, 764774. 10.3389/FPHAR.2022.764774 35153773 PMC8830515

[B9] Ihama Y. Fukasawa M. Ninomiya K. Kawakami Y. Nagai T. Fuke C. (2014). Anaphylactic shock caused by sting of crown-of-thorns starfish (Acanthaster planci). Forensic Sci. Int. 236, e5–e8. 10.1016/j.forsciint.2014.01.001 24491916

[B10] Jain A. Sarsaiya S. Wu Q. Shi J. Lu Y. (2019). New insights and rethinking of cinnabar for chemical and its pharmacological dynamics. Bioengineered 10 (1), 353–364. 10.1080/21655979.2019.1652491 31431119 PMC6738451

[B11] Jalili J. Askeroglu U. Alleyne B. Guyuron B. (2013). Herbal products that may contribute to hypertension. Plast. Reconstr. Surg. 131 (1), 168–173. 10.1097/PRS.0b013e318272f1bb 23271526

[B12] Jeon S. Jeong W. Park J. You Y. Ahn H. Kim S. (2020). Clinical relationship between blood concentration and clinical symptoms in aconitine intoxication. Am. J. Emerg. Med. 40, 184–187. 10.1016/J.AJEM.2020.11.005 33243534

[B13] Karturi S. P. Gudmundsson H. Akhtar M. Jahangir A. Choudhuri I. (2016). Spectrum of cardiac manifestations from aconitine poisoning. HeartRhythm Case Rep. 2 (5), 415–420. 10.1016/j.hrcr.2016.05.007 28491724 PMC5419964

[B14] Li S. Liu Y. (2018). Literature analysis of allergic shock induced by Niuhuang Jiedu Tablets(pills). China J. Pharm. Econ. 13 (07), 122–124. 10.12010/j.issn.1673-5846.2018.07.038

[B15] Li S. Yu L. Shi Q. Liu Y. Zhang Y. Wang S. (2022). An insight into current advances on pharmacology, pharmacokinetics, toxicity and detoxification of aconitine. Biomed. Pharmacother. 151, 113115. 10.1016/J.BIOPHA.2022.113115 35605296

[B16] Luo Y. Lv L. Liu G. Zhang M. Xu W. (2018). Analysis of adverse drug reactions of Zhonghuadieda pills in 204 cases. Pop. Sci. Technol. 20 (09), 63–65. 10.3969/j.issn.1008-1151.2018.09.020

[B17] Ma L. Jin W. (1994). Excessive Zixue powder causes death. Chin. J. Pediatr. (02), 122.

[B18] Mani J. Rutz J. Maxeiner S. Juengel E. Bon D. Roos F. (2019). Cyanide and lactate levels in patients during chronic oral amygdalin intake followed by intravenous amygdalin administration. Complement. Ther. Med. 43, 295–299. 10.1016/j.ctim.2019.03.002 30935547

[B19] National Center for Adverse Drug Reaction Monitoring (NCADRM) (2018). Annual report for national adverse drug reaction Monitoring. Available at: https://www.nmpa.gov.cn/xxgk/ggtg/ypggtg/ypqtggtg/20191018151301540.html .

[B20] National Center for Adverse Drug Reaction Monitoring (NCADRM) (2019). Annual report for national adverse drug reaction monitoring. Available at: https://www.nmpa.gov.cn/xxgk/yjjsh/ypblfytb/20200413094901811.html .

[B21] National Center for Adverse Drug Reaction Monitoring (NCADRM) (2020). Annual report for national adverse drug reaction Monitoring. Available at: https://www.nmpa.gov.cn/directory/web/nmpa/xxgk/fgwj/gzwj/gzwjyp/20210325170127199.html .

[B22] Patel K. Gin A. Scardamaglia L. (2021). Palmoplantar keratosis caused by arsenic toxicity. Med. J. Aust. 214 (6), 258. 10.5694/mja2.50965 33629396

[B23] Ren S. Cao Y. Zhang X. Jiao S. Qian S. Liu P. (2014). Cephalosporin induced disulfiram-like reaction: a retrospective review of 78 cases. Int. Surg. 99 (2), 142–146. 10.9738/INTSURG-D-13-00086.1 24670024 PMC3968840

[B24] Su F. Sun Y. Zhu W. Bai C. Zhang W. Luo Y. (2022). A comprehensive review of research progress on the genus Arisaema: Botany, uses, phytochemistry, pharmacology, toxicity and pharmacokinetics. J. Ethnopharmacol. 285, 114798. 10.1016/J.JEP.2021.114798 34780984

[B25] Tian J. Liang A. Zhu X. Zhao Y. Yi Y. Li C. (2019). Advances in the safety evaluation of mineral medicines–Cinnabar and Realgar. World J. Tradit. Chin. Med. 5 (3), 164–172. 10.4103/wjtcm.wjtcm_1_19

[B26] Tong X. Fang M. Gao H. Jia T. Shang H. Song Y. (2021). Major scientific and Engineering problems of traditional Chinese medicine in 2021. J. Tradit. Chin. Med. 62 (11), 921–929. 10.13288/j.11-2166/r.2021.11.001

[B27] Tong Y. Zhang L. Yang J. Yang L. Wang Y. Qu J. (2010). Retrospective study of adverse reactions of Niuhuang Jiedu tablet (pill) and risk control based on literature analysis. China J. Chin. Mater. Med. 35 (10), 1342–1345. 10.4268/cjcmm20101027 20707211

[B28] Vergara-Gerónimo C. A. León Del Río A. Rodríguez-Dorantes M. Ostrosky-Wegman P. Salazar A. M. (2021). Arsenic-protein interactions as a mechanism of arsenic toxicity. Toxicol. Appl. Pharmacol. 431, 115738. 10.1016/J.TAAP.2021.115738 34619159

[B29] Wang B. Zhao Y. Zhang Y. Ge L. Mao Y. (2016). Observation on curative effect of Xiaoer Huadu powder in treatment of acute upper respiratory tract infection in children. Shanxi Med. J. 45 (24), 2933–2935. 10.3969/j.issn.0253-9926.2016.24.038

[B30] Xin J. (2017). Analysis of adverse reactions induced by Xiaojin pills and Xiaojin capsules. Cardiovasc. Dis. Electron J. Integr. Tradit. Chin. West. Med. 5 (20), 138–139. 10.16282/j.cnki.cn11-9336/r.2017.20.102

[B31] Xu P. Song Y. Feng B. Zeng Q. Shan B. Liu K. (2018). Multi-component profiles through the blood-brain barrier in rat after oral administration of over-the-counter drug Keke capsule by ultra-performance liquid chromatography/quadrupole-time-of-flight MS^E^ method. Biomed. Chromatogr. 33 (1), e4380. 10.1002/bmc.4380 30178888

[B32] Yang C. (1991). Report on 1 case of neonatal poisoning death caused by Bingpeng powder. Chin. J. Integr. Tradit. West. Med. (03), 146.

[B33] Yang S. (1997). One case of death by intravenous infusion of Huoxiangzhengqi water. Forensic. Sci. and Tech. (03), 44. 10.16467/j.1008-3650.1997.03.025

[B34] Yang S. Lv J. Wang L. Xie Y. (2022). Systematic review and meta-analysis of efficacy and safety of Binghuang Fule ointment in treatment of eczema. China J. Chin. Mater. Med. 47 (10), 2802–2810. 10.19540/j.cnki.cjcmm.20220215.501 35718500

[B35] Yang X. Xiao C. Zhang K. (2015). Progress in clinical application and pharmacological action of ephedra. Chin. Arch. Tradit. Chin. Med. 33 (12), 2877. 10.13193/j.issn.1673-7717.2015.12.015

[B36] Yi Y. Li C. Y. Zhao Y. Zhang Y. S. Han J. Y. Wang L. M. (2018). Safety of animal traditional Chinese medicine (TCM) injections. China J. Chin. Mater. Med. 43 (22), 4391–4396. 10.19540/j.cnki.cjcmm.20180711.001 30593229

[B37] Zhang M. Lin Z. Zhu G. Li J. Zheng Y. (2008). 247 cases of chronic gastritis with dyspepsia treated by quadruple therapy combined with Xiangsha Yangwei pill. Chin. J. Integr. Tradit. West. Med. Dig. (05), 341–342.

[B38] Zhang N. Shi N. Li S. Liu G. Han Y. Liu L. (2020). A retrospective study on the use of Chinese patent medicine in 24 medical institutions for COVID-19 in China. Front. Pharmacol. 11, 574562. 10.3389/fphar.2020.574562 33776751 PMC7990099

[B39] Zhou D. (2009). Clinical comparative study on treatment of migraine by high dose of Ershiwuwei Shanhu pills. Sichuan Med. J. 30 (05), 657–659. 10.16252/j.cnki.issn1004-0501-2009.05.005

