# Supplementary material for: ‘Risk-benefit’ assessment for comprehensive safety evaluation of Chinese patent medicines containing four common toxic ingredients: an analysis of clinical risk factors
Source: Front Pharmacol. 2024 Aug 23;15:1324509. doi: 10.3389/fphar.2024.1324509 (PMC11377351; doi:10.3389/fphar.2024.1324509)
Supplement: Supplementary file 1 [file Table1.pdf]

Table legends

Table S1. CHMs with different toxicity levels in *Chinese Pharmacopoeia* (2020)

Table S2. The main types of toxic ingredients, toxic CHMs, and the corresponding quantity of CPMs

Table S3. Classification of CPMs with different toxic ingredients

Table S4. System/organ involved in ADEs of CPMs containing toxic ingredients

Table S5. Systems/organs and clinical manifestations involved in ADEs related to CPMs containing hydrogen cyanide

Table S6. System/organ involved and clinical manifestations of ADEs related to CPMs containing mineral composition

Table S7. System/organ involved and clinical manifestations of ADEs related to CPMs containing Araceae metabolites

Table S8. System/organ involved and clinical manifestations of ADEs related to CPMs containing diester aconitine metabolites

Table S1. CHMs with different toxicity levels in *Chinese Pharmacopoeia* (2020)

Toxicity classification	Toxic CHMs
Strong Toxicity	Radix Aconiti Communis, Strychnos, Strychnine Powder, Hyoscyamus Niger, Croton, Croton Cream, Red Halophiles Iron Oxide, Chinese Azalea Flower, Kusnezoff Monkshood Root, Cantharides
Toxicity	Barberry, Dried Lacquer, Goldenlarch Bark, Tonkin Sophora Root, Caper Euphorbia Seed, Caper Euphorbia Seed Cream, Radix Aconiti Preparata, Reddish Jackinthepulpit Rhizome, Processed Rhizoma Arisaematis, Cochinchina Momordica Seed, Kansui Euphorbia Root, Common Cruculigo Rhizome, Giant Typhonium Rhizome, Ginkgo Seed, Greater Celandine, Ternate Pinellia Rhizome, Cinnabar, Funneled Physochlaina Root, Scorpion, Lilac Daphne Immature Flower, Siberian Cocklebur Fruit, Radde Anemone Rhizome, Radix Aconiti Carmichaeli, Chinaberry-Tree Bark, Little Multibanded Krait, Peking Euphorbia Root, Prepared Aconite Root, Lobedleaf Pharbitis Seed, Mercurous Chloride, Chinese Silkvine Root-Bark, Flora Datura, Wingedtooth Laggera Herb, Chinese Stellera Root, Antifebrile Dichroa Root, Indian Pokeweed Root, Sulphur, Realgar, Castorbean Seed, Centipede, Opium Poppy Pericarp, Long-Noded Pit Viper, Toad
Small Toxicity	Obtuseleaf Erycibe Stem, Common Jasminorange, Eupolyphaga Steleophaga, Chinese Honeylocust, Chinaberry Fruit, Himalayan Mayapple, Feiyangcao, Leech, Argy Wormwood Leaf, Asiatic Moonseed Rhizome, Difengpi, Knoxia Root, Pricklyash, Medicinal Evodia Fruit, India Quassiawood, Bitter Almond, Psammosilene Tunicoides, Aconitum Leaf, Wild Carrot Fruit, Java Brucea Fruit, Yunnan Manyleaf Paris Rhizome, Semen Impatientis, Fructus Cnidii, Chinese Honeylocust Fruit, Basket Fern Rhizome, Dryopteridis Crassirhizomatis Rhizoma Carbonisatum, Japanese Flowering Fern Rhizome, Puncturevine, Entada Phaseoloides, Common Carpesium Fruit, Pterocephalus Hookeri

Table S2. The main types of toxic ingredients, toxic CHMs, and the corresponding quantity of CPMs

Toxic ingredients	Quantity of CHMs	Name of CHMs	Quantity of CPMs	Total	Proportion
Araceae metabolites	4	Pinellia ternata (Thunb.) Makino [Araceae; Pinelliae Rhizoma; Banxia]	96	116	29.3%
		Arisaema heterophyllum Blume [Araceae; Arisaematis Rhizoma Preparatum; Zhitiannanxing]	14		
		Sauromatum giganteum (Engl.) Cusimano & Hett. [Araceae; Typhonii Rhizoma; Baifuzi]	8		
		Arisaema erubescens (Wall.) Schott [Araceae; Arisaematis Rhizoma; Tiannanxing]	7		
hydrogen cyanide	2	Prunus armeniaca L. [Rosaceae; Armeniacae Semen Amarum; Kuxingren]	96	97	24.5%
		Ginkgo biloba L. [Ginkgoaceae；Ginkgo Semen; Yinxingren]	1		
Mineral composition	5	Cinnabar [Sulfides; Cinnabaris; Zhusha]	71	87	22.0%
		Red orpiment [Sulfides; Realgar; Xionghuang]	38		
		Hongfen [Hydrargyri Oxydum Rubrum]	3		
		Qingfen [Calomelas]	2		
		Liuhuang [Sulfur]	1		
diester aconitine metabolites	5	Sauromatum giganteum (Engl.) Cusimano & Hett. [Araceae; Typhonii Rhizoma; Baifuzi]	24	66	16.7%
		Aconitum kusnezoffii Rchb. [Ranunculaceae; Aconti Kusnezoffii Radix Cocta; Zhicaowu]	23		
		Aconitum carmichaelii Debeaux [Ranunculaceae;	22		

		Aconiti Radix Cocta; Zhiwutou]			
		Aconitum carmichaelii Debeaux [Ranunculaceae; Aconiti Radix; Shengwutou]	12		
		Aconitum kusnezoffii Rchb. [Ranunculaceae; Aconiti Kusnezoffii Radix; Caowu]	11		
Cardiac glycosides	2	Toad skin Secretion cake [Bufonidae; Bufonis Venenum; Chansu]	14	21	5.3%
		Periploca sepium Bunge [Apocynaceae; Periplocae Cortex; Xiangjiapi]	7		
Scopolamine	4	Datura metel L. [Solanaceae; Daturae flos; Yangjinhua]	6	9	2.3%
		Hyoscyamus niger L. [Solanaceae; Hyoscyami Semen; Tianxianzi]	1		
		Erycibe obtusifolia Benth. [Convolvulaceae; Erycibes Caulis; Dinggongteng]	1		
		Physochlaina infundibularis Kuang [Solanaceae;Physochlainae Radix; Huashanshen]	1		

Table S3. Classification of CPMs with different toxic ingredients

Type of CPMs	Name of CPMs
CPMs containing Araceae metabolites	Yigan Yiqi Jieyu granule, Xiaoer Baibu Zhike syrup, Xiaoer Zhibao pill, Yuzhen powder, Erchen pill, Wuhu powder, Baolong pill, Baikejing syrup, Xiaoer Chiqiao Qingre granule, Niuhuang Huadu tablet, Fufang Qianzheng ointment, Shangjie ointment, Zhisou Huatan pill, Xinnaojing tablets, Qingxuan Zhitan pills, Ruyi Jinhuang powder, Shaoyang Ganmao granules, Miaoling pill, Xingnao Zaizao capsule, Qushang Xiaozhong Tincture, Niuhuang Zhenjing pill, Guogong Wine, Tongqiao Erlong pill, Huoxue Zhitong ointment, Baokening granule, Xinsuning capsule, Dingchuan ointment, Zhengqi tablet, Guci pill, Jiawei Huoxiang Zhengqi Soft capsule, Guci Xiaotong tablet, Zhuli Datan pill, Baochi powder, Yixian pill, Zhuifeng Tougu pill, Shensu pill, Jintongxiao Tincture, Kechuanshun pill, Xiangshayangwei pill, Xiangshayangwei pill (concentrated pill), Xiangshayangwei granule, Baohe pill, Baohe pill (Water pill), Baohe tablet, Baohe granule, Guishao Zhenxian tablet, Tongxuan Lifei pill, Tongxuan Lifei tablet, Tongxuanlifei granule, Tongxuanlifei capsule, Qingqi Huatan pill, Qingning pill, Jieji Ningsou pill, Ertong Qingfei pill, Ertong Qingre Daozhi pill, Xiaoer Jindan tablet, Xiaoer Xiangju pill, Xiaoqinglong Mixture, Xiaoqinglong granule, Xiaochaihu tablet, Xiaochaihu effervescent tablet, Xiaochaihu capsule, Xiaochaihu granule, Zhike Juhong oral liquid, Zhike Juhong pill, Fenghan Kesou pill, Fenghan Kesou granule, Liujunzi pill, Shuanghu Qinggan granule, Sizheng pill, Banxia Tianma pill, Dalitong granule, Qingyu Piwen pill, Suzi Jiangqi pill, Chunyang Zhengqi pill, Shenshuaining capsule, Hezhong Lipi pill, Hewei Zhixie capsule, Jinpu capsule, Jinsang Liyan pill, Baizi Yangxin pill, Baizi Yangxin tablet, Weilikang tablet, Xiangsha Liujun pill, Xiangsha Hezhong pill, Fufang Chuanbeijing tablet, Fufang Xianzhuli liquid, Hengzhi Kechuan capsule, Guilong Kechuanning capsule, Guilong Kechuanning granule, Chaihu Shugan pill, Naoliqing pill, Naoliqing capsule, Yishen Huashi granule, Xiaoxuan Zhiyun tablet, Tiaojing pill, Tiaowei Xiaozhi pill, Tianmeng capsule, Tianmeng oral liquid (Tianmeng Mixture), Qingfei Huatan pill, Yueju Erchen pill, Shuzheng tablet, Shushi Ganmao granule, Shugan Pingwei pill, Jieyu Anshen granule, Xixian Tongshuan pill, Xixian Tongshuan capsule, Juhong tablet, Juhong capsule, Juhong granule, Juhong pill, Juhong Tanke liquid, Huoxiang Zhengqi oral liquid, Huoxiang Zhengqi liquid, Huoxiang Zhengqi Soft capsule, Huoxiang Zhengqi dropping pill.
CPMs containing mineral composition	Yinianjin powder, Yinianjin capsule, Ershiwuwei Songshi pill, Ershiwuwei Shanhu pill, Shixiang Fansheng pill, Qizhen pill, Qili capsule, Qili Power, Renshen Zaizao pill, Wanshi Niuhuang Qingxin pill, Xiaoer Baishou pill, Xiaoer Zhibao pill, Xiaoer Jindan tablet, Xiaoer Feireping capsule, Xiaoer Jingfeng Power, Xiaoerqingre tablet, Xiaoerjiere pill, Tianwang Buxin pill, Tianwang Buxin pill (concentrated pill), Yatong Yili pill, Niuhuang Qianjin powder, Niuhuang Baolong pill, Niuhuangjingnao tablet, Niu Huang

	Qingxin pill (Ju Fang), Niu Huang Qinggong pill, Niuhuang Zhenjing pill, Qitong pill, Renqing Mangjue, Renqing Changjue, Xinnaojing tablet, Pinggan Shuluo pill, Guashuang Tuireling capsule, Zaizao pill, Shangke Jiegu tablet, Bingpeng powder, Qingyu Biwen pill, Angong Niuhuang pill, Angong Niuhuang powder, Annao pill, Annao tablet, Hongling Power, Suhexiang pill, Yixian pill, Kangshuan Zaizao pill, Bushen Yinao pill, Bushen Yinao tablet, Jufang Zhibao powder, Miaoling pill, Chunyang Zhengqi pill, Baolong pill, Zhoushi Huisheng pill, Baizi Yangxin pill, Baizi Yangxin tablet, Xiangsu Zhengwei pill, Fufang Niuhuang Xiaoyan capsule, Baochi Power, Suxiao Niuhuang pill, Yiyuan powder, Tongbi tablet, Tongbi capsule, Meihua Dianshe pill, Qingxie pill, Huopo Baolong pill, Zijin powder, Zixue powder, Shuzheng tablet, Dieda Qili tablet, Shugan pill, Shugan pill (Concentrated pill), Shayao, Biwen powder, Xiaoer Huadu powder, Niuhuang Zhibao pill, Niuhuang Xiaoyan tablet, Niu Huang Jiedu pill, Niu Huang Jiedu tablet, Niu Huang Jiedu Soft capsule, Niu Huang Jiedu capsule, Liuying pill, Kelisha capsule, Awei Huapi ointment, Yujin Yinxie tablet, Zhuhuang Chuihou powder, Jiuyi powder, Jiusheng powder, Badu ointment, Binghuangfule ointment
CPMs containing hydrogen cyanide	Ermuansou pill, Ertong Qingfei pill, Ergan Tuirening oral liquid, Jiusheng powder, Sanao tablet, Dahuang Zhechong pill, Wanling Wuxiang ointment, Xiaoer Baibu Zhike syrup, Xiaoer Feirekechuan oral liquid, Xiaoer Kechuanling oral liquid, Xiaoer Kechuan granule, Xiaoerqingfei Zhike tablet, Xiaoerqingfei Huatan oral liquid, Xiaoer Qingre Zhike Mixture (Xiaoer Qingre Zhike oral liquid). Xiaoer Ganmaoning syrup, Zhike Juhong oral liquid, Zhike Juhong pill, Zhichuanling Injection, Zhisou Huatan pill, Zhisou Dingchuan oral liquid, Niuhuang Qingxin pill (Ju Fang), Huazheng Huisheng tablet, Fenghan Kesou pill, Fenghan Kesou granule, Shihu Yeguang pill, Sifangwei tablet, Sifangwei capsule, Waigan Fenghan granule, Fengliaoxing Fengshidieda Wine, Baikejing syrup, Ruyi Dingchuan tablet, Qihuang Tongmi Soft capsule, Keke tablet, Xingsu Zhike Qral liquid, Xingsu Zhike granule, Xingsu Zhike syrup, Lianhua Qingwen tablet, Lianhua Qingwen capsule, Lianhua Qingwen granule, Biaoshi Ganmao granule, Biaoxu Ganmao granule, Kugan granule, Shenyan Jiere tablet, Jinbei Tankeqing granule, Jinlian Qingre granule, Jinsang Kaiyin pill, Jinsang Kaiyin granule, Baokening granule, Kechuanning oral liquid, Kechuanshun pill, Fufang Yigan pill, Fufang Haqing tablet, Yangshen Baofei pill, Guilong Kechuanning capsule, Guilong Kechuanning granule, Chaiyin oral liquid, Shema oral liquid, Yifei Qinghua ointment, Runfei Zhisou pill, Tongyou Runzao pill, Tongxuan Lifei pill, Tongxuan Lifei tablet, Tongxuan Lifei capsule, Tongxuan Lifei granule, Sangjiang Ganmao tablet, Sangju Ganmao pill, Sangju Ganmao tablet, Sangju Ganmao Mixture, Yinhuang Qingfei capsule, Maren pill, Maren Runchang pill, Maren Zipi pill, Lingyang Qingfei pill, Lingyang Qingfei granule, Qingqi Huatan pill, Qingfei Huatan pill, Qingfei Xiaoyan pill, Qingxuan Zhike

	granule, Hupo Huanjing pill, Tingbei capsule, Shushi Ganmao granule, Gejie Dingchuan pill, Gejie Dingchuan capsule, Ganmao Zhike granule, Ganmao Qingre oral liquid, Ganmao Qingre chewable tablet, Ganmao Qingre capsule, Ganmao Qingre granule, Jieji Ningsou pill, Juhong pill, Juhong tablet, Juhong capsule, Juhong granule, Juhong Huatan pill, Juhong Tanke liquid, Lusika pill, Chushi Baidai pill
CPMs containing diester aconitine metabolites	Renshen Zaizao pill, Tianma pill, Wumei pill, Shengbai Mixture (Shengbai oral liquid), Zaizao pill, Yanghe Jiejing ointment, Fuzi Lizhong pill, Fuzi Lizhong tablet, Fugui Gutong tablet, Fugui Gutong capsule, Fuguigutong granule, Yixin pill, Guben Tongxue granule, Dingchuan ointment, Shenfu Qiangxin pill, Qianlieshu pill, Jisheng Shenqi pill, Guifu Dihuang oral liquid, Guifu Dihuang pill, Guifu Dihuang capsule, Yishenling granule, Tongbi tablet, Tongbi capsule, Weidakang oral liquid, Sanqi Shangyao tablet, Sanqi Shangyao capsule, Sanqi Shangyao granule, Sanqi Xueshangning capsule, Xiaojin pill, Xiaojin tablet, Xiaojin capsule, Xiaohuoluo pill, Mugua pill, Fengshi Gutong tablet, Fengshi Gutong capsule, Fenghan Shuangliguai tablet, Shenjin Huoluo pill, Guci pill, Guci Xiaotong tablet, Fufang Xiatianwu tablet, Zhuifeng Tougu pill, Qufeng Zhitong pill, Qufeng Zhitong tablet, Qufeng Zhitong capsule, Qufengshujin pill, Jintongxiao Tincture, Qiangli Tianma Duzhong pill, Zhonghua Dieda pill, Qingyu Piwen pill, Guyouling Chaji, Fufang Yangjiao tablet, Huoxue Zhuangjin pill, Shexiang Fengshi capsule, Wanling Wuxiang ointment, Tianhe Zhuifeng ointment, Shaolin Fengshi Dieda ointment, Shangshi Zhitong ointment, Anyang Jingzhi ointment, Awei Huapi ointment, Goupi ointment, Medicinal moxa Stick, Shexiang Dieda Fengshi ointment, Shexiang Zhentong ointment, Qushang Xiaozhong Tincture, Zhenggu liquid, Dieda Zhentong ointment

Table S4. System/organ involved in ADEs of CPMs containing toxic ingredients

ADEs Systems	CPMs containing hydrogen cyanide		CPMs containing Araceae metabolites		CPMs containing mineral composition		CPMs containing diester aconitine metabolites	
	Frequency	Proportion	Frequency	Proportion	Frequency	Proportion	Frequency	Proportion
Digestive system	337	50.8%	185	34.4%	202	32.6%	314	35.6%
Skin and its appendage	223	33.6%	33	6.1%	138	22.3%	338	38.4%
Allergic reaction	17	2.6%	50	9.3%	68	11.0%	93	10.6%
Urinary system	2	0.3%	18	3.3%	35	5.7%	5	0.6%
Respiratory system	5	0.8%	1	0.2%	32	5.2%	2	0.2%
Nervous system	51	7.7%	64	11.9%	25	4.0%	35	4.0%
Systemic response	4	0.6%	62	11.5%	23	3.7%	5	0.6%
Muscle and skeletal system	0	0.0%	0	0.0%	22	3.6%	8	0.9%
Toxic reaction	0	0.0%	10	1.9%	21	3.4%	7	0.8%
Liver injury	4	0.6%	3	0.6%	16	2.6%	14	1.6%
Addiction	0	0.0%	0	0.0%	11	1.8%	0	0.0%
Circulatory system	17	2.6%	108	20.1%	10	1.6%	41	4.7%
Blood system	1	0.2%	0	0.0%	8	1.3%	2	0.2%
Ear, Nose, and Throat (ENT) reaction	1	0.2%	2	0.4%	4	0.6%	7	0.8%
Reproductive system	1	0.2%	0	0.0%	1	0.2%	10	1.1%
Death	0	0.0%	1	0.2%	2	0.3%	0	0.0%
Other	0	0.0%	1	0.2%	1	0.2%	0	0.0%
Total	663	100%	538	100%	619	100%	881	100%

Table S5. Systems/organs and clinical manifestations involved in ADEs related to CPMs containing hydrogen cyanide

CPMs containing hydrogen cyanide	Involved system/organ	Frequency	Clinical manifestations of ADE (cases)	Total
Keke tablet	Skin and its appendage	210	Rash (140), Pruritus (62), Red rash (2), Measles (2), Redness (2), Dermatitis (1), Itch (1)	312
	Digestive system	52	Nausea (20), Vomiting (11), Abdominal pain (9), Diarrhea (4), Dry mouth (2), Epigastric discomfort (1), Loss of appetite (1), Bitter taste in the mouth (2), Thirst (3), Abdominal distension (3), Constipation (4)	
	Nervous system	29	Dizziness (20), Headache (6), Vertigo (1), Insomnia (2)	
	Circulatory system	12	Chest tightness (7), Elevated blood pressure (2), Arrhythmia (1), Palpitation (1), Hyperhidrosis (1)	
	Respiratory system	5	Panic (1), Wheezing (2), Dyspnea (3)	
	Systemic reaction	2	Fatigue (2)	
	Liver damage	1	Elevated transaminases (1)	
	ENT reaction	1	Tinnitus (1)	
Lianhua Qingwen tablet (capsule, granule)	Digestive system	179	Gastrointestinal discomfort (78), Diarrhea (54), Nausea and vomiting (46), Thirst (1)	214
	Nervous system	19	Somnolence (12) Dizziness and headache (7)	
	Allergic reaction	14		
	Urinary system	1	Acute urinary retention (1)	
	Liver damage	1		
Dahuang Zhechong pill	Digestive system	58	Abdominal pain, Increased stool frequency, loose stools (42)<br>Diarrhea (16)	62
	Skin and its appendage	2	Acne (1), Rash (2)	
	Reproductive system	1	Vaginal dryness (1)	
	Circulatory system	1	Low Heat (1)	
Maren pill	Digestive system	11	Abdominal pain (4), Gastrointestinal discomfort (4), Loose stool (2), Diarrhea (1)	16

	Circulatory system	4	Pulse rate (2), Arrhythmia (2)	
	Allergic reaction	1		
Xiaoer Feire Kechuan oral liquid	Digestive system	11	Diarrhea (4), Nausea and vomiting (4), Increased stool frequency, Loose stool (3)	11
Maren Runchang pill	Digestive system	9	Diarrhea (6), Abdominal discomfort (1), Abdominal pain (1), Loose stool (1)	9
Xiaoer Kechuanling oral liquid	Respiratory system	2	Cough (1), Influenza (1)	5
	Liver damage	2	Abnormal AST (1), Abnormal ALT (1)	
	Systemic reaction	1	Fever (1)	
Chaiyin oral liquid	Digestive system	2	Nausea and vomiting (2)	4
	Blood system	1	Leukopenia (1)	
	Skin and its appendage	1	Rash (1)	
Juhong Tanke liquid	Digestive system	4	Mild abdominal distension (1), Stomach discomfort (1), Mild stomach pain (1), Moderate stomach pain (2)	4
Lingyang Qingfei granule	Digestive system	2	Nausea (1), Vomiting (1)	3
	Skin and its appendage	1	Itchy skin (1)	
Qingxuan Zhike granule	Digestive system	3	Diarrhea (3)	3
Ganmao Qingre granule	Nervous system	2	Somnolence (1), Somnolence with dizziness (1)	3
	Systemic reaction	1	Fatigue (1)	
Sanao tablet	Urinary system	1	Water retention (1)	2
	Nervous system	1	Neuropsychiatric symptom (1)	
Guilong Kechuanning capsule	Allergic reaction	2		2
Gejie Dingchuan pill	Digestive system	1	Upper gastrointestinal bleeding (1)	1
Jinlian Qingre granule	Digestive system	1	Dull pain in the epigastric region (1)	1

Table S6. System/organ involved and clinical manifestations of ADEs related to CPMs containing mineral composition

CPMs containing mineral composition	Involved system/organ	Frequency	Clinical manifestations of ADE (cases)	Total
Niuhuang Jiedu pill (tablet)	Allergic reaction	53	Anaphylaxis (36), Anaphylactic shock (14), Allergic herpes simplex (1), Skin allergy (1), Allergic glossitis (1)	244
	Urinary system	32	Urinary system reaction (28), Cystitis (1), Hemorrhagic cystitis (1), Hematuria (2)	
	Skin and its appendage	38	Drug eruption (18), Fixed drug eruption (8), Urticaria (4), Fixed erythema (1), Scrotal red macula with ulceration and exudation (1), Generalized urticaria (1), Erythema of limbs with pruritus (1), Local skin flushing (1), Scarlatiniform rash (1), Exfoliative dermatitis type drug eruption (1), Dermatitis medicamentosa (1)	
	Digestive system	36	Digestive system reaction (35), Gastrointestinal bleeding (1)	
	Respiratory system	32	Respiratory response (31), Bronchial asthma (1)	
	Nervous system	20	Nervous system (19), Nausea, dizziness, fatigue (1)	
	Toxic reaction	11	Chronic arsenic poisoning (8), Toxic reaction (3)	
	Addiction	11		
	Liver damage	5	Occlusion of hepatic venules (2), Drug-induced liver disease (1), Elevated transaminases (1), Impaired liver function (1)	
	Blood system	4	Pure red cell aplasia (1), Thrombocytopenia (1), Hematological reaction (1), Hemolytic anemia (1)	
	ENT reaction	1	Epistaxis (1)	
	Other	1	Stevens-Johnson syndrome (1)	
Binghuang Fule ointment	Skin and its appendage	91	Burning sensation (12), Local irritation (8), Pigmentation (2), Skin erythema (4), Burning sensation and increased itching (7), Dry skin, fine scales (10), Facial erythema, burning sensation (5), Mild erythema, desquamation (8), Mild tingling (1), Mild dryness and burning sensation (5), Local redness, mild pain	97

			(6), Local pain, burning sensation and erythema (12), Desquamation (1), Increased chapped skin (3), Dry, wrinkled skin with small scales (4), Enlarged skin lesions with marked erythema and increased itching (2), Skin flushing (1)	
	Digestive system	4	Nausea (2), Vomiting (2)	
	Nervous system	2	Dizziness (2)	
Shangke Jiegu tablet	Systemic reaction	23	Fever (23)	52
	Muscle and skeletal system	20	Sore (20)	
	Allergic reaction	4	Allergic rash (4)	
	Skin and its appendage	3	Local skin flushing (2), itching (1)	
	Digestive system	1	Gastrointestinal discomfort (1)	
	Reproductive system	1	Vaginal bleeding (1)	
Bingpeng powder	Digestive system	46	Increased saliva (17), Nausea (11), Nausea and vomiting (1), Mouth discomfort (9), Abdominal distension (6), Severe abdominal pain (1), Multiple colon perforations (1)	49
	Allergic reaction	2	Anaphylactic shock (1), Allergic stomatitis (1)	
	Death	1		
Ershiwuwei Shanhu pill	Digestive system	33	Nausea, gastric discomfort (3), Gastrointestinal discomfort (30)	33
Angong Niuhuang pill	Digestive system	19	Diarrhea (5), Vomiting (1), Abdominal distension (3), Loose stools (2), Anorexia, nausea (8)	26
	Circulatory system	4	Hypertensive Encephalopathy (1), Hypothermia (3)	
	Allergic reaction	2		
	Muscle and skeletal system	1	Rhabdomyolysis (1)	
Guashuang Tuireling capsule	Digestive system	18	Loose stool (7), Nausea (7), Vomiting (4)	18
Xiaoer Huadu powder	Digestive system	12	Nausea and vomiting (7), Diarrhea (5)	15

Annao tablet	Skin and its appendage	3	Rash (3)	11
	Digestive system	4	Diarrhea (2), Nausea, vomiting, gastrointestinal discomfort, diarrhea (2)	
	Nervous system	1	Headache and swelling (1)	
	Liver damage	5	Liver Injury (5)	
	Urinary system	1	Hematuria (1)	
Yujin Yinxie tablet	Digestive system	10	Diarrhea (6), Gastrointestinal reaction (4)	11
	ENT reaction	1	Nosebleed (1)	
Renqing Chang jue	Blood system	4	Leukopenia (4)	10
	Digestive system	3	Nausea and vomiting (3)	
	Circulatory system	3	Hypotension (3)	
Tongbi capsule	Nervous system	2	Headache (1), Dizziness and fatigue (1)	10
	Skin and its appendage	2	Rash (1), Itchy skin (1)	
	Digestive system	1	Stomach nausea (1)	
	Circulatory system	1	Abnormal ECG (1)	
	Urinary system	1	Urinary occult blood (1)	
	Muscle and skeletal system	1	Low back pain (1)	
	ENT reaction	2	Muchi (2)	
Annao pill	Digestive system	2	Nausea (1), Thirst (1)	8
	Liver and kidney damage	6	Hepatic and renal dysfunction (6)	
Suhexiang pill	Toxic reaction	6	Poisoning (6)	7
	Allergic reaction	1	Anaphylactic shock (1)	
Yinianjin	Digestive system	6	Diarrhea (6)	6
Dieda Qili tablet	Digestive system	5	Gastric discomfort (5)	5
Niuhuang Xiaoyan tablet	Toxic reaction	4	Poisoning (4)	5
	Skin and its appendage	1	Urticaria (1)	
Qili powder	Allergic reaction	3	Allergic dermatitis (3)	3
Qili capsule	Allergic reaction	1	Allergic drug eruption (1)	2

	Digestive system	1	Constipation (1)	
Liuying pill	Digestive system	1	Gastrointestinal bleeding (1)	2
	Allergic reaction	1	Allergic dermatitis (1)	
Tongbi tablet	Circulatory system	1	Palpitation with numbness of lip and tongue (1)	1
Zijin powder	Allergic reaction	1	Allergic rash (1)	1
Zixue powder	Death	1		1
Ershiwuwei Songshi pill	Urinary system	1	Severe renal impairment (1)	1
Baizi Yangxin pill	Circulatory system	1	AV Block (1)	1

Table S7. System/organ involved and clinical manifestations of ADEs related to CPMs containing Araceae metabolites

CPMs containing Araceae metabolites	Involved system/organ	Frequency	Clinical manifestations of ADE (cases)	Total
Huoxiang Zhengqi liquid	Circulatory system	79	Skin flushing (74), Supraventricular tachycardia (4), Facial flushing and palpitation (1)	232
	Systemic reaction	29	Disulfiram-like reaction (16), Infantile convulsions (10), Drunk-like anaphylaxis (2), Coma (1)	
	Allergic reaction	36	Anaphylaxis (22), Anaphylactic shock (7), Anaphylactic rash (2), Henoch-Schonlein purpura (2), Allergic asthma (2), Allergic dermatitis (1)	
	Digestive system	47	Bitter taste (36), Gastrointestinal bleeding (2), Intestinal obstruction (2), Superficial gastritis (1), Hypoglycemia (6)	
	Nervous system	25	Dizziness (23), Psychotic disorder (1), Involuntary movements (1)	
	Toxic reaction	10	Alcohol poisoning (3), poisoning (7)	
	Skin and its appendage	5	Urticaria-like drug eruption (4), Dermatomyositis (1)	
	Death	1		
Tianmeng capsule	Nervous system	37	Insomnia (17), Dizziness (10), Somnolence (5), Emotional depression (5)	98
	Systemic reaction	32	Asthenia (27), Increased activity (2), Obesity (3)	
	Circulatory system	17	Tachycardia (13), Palpitation (2), Hypotension (2)	
	Digestive system	10	Dry mouth (4), Nausea and vomiting (2), Anorexia (2), Constipation (2)	
	ENT reaction	2	Blurred vision (1), Nasal congestion (1)	
Xiangsha Yangwei pill	Digestive system	40	Loose stool (15), Increased stool frequency (13), Abdominal pain or aggravation of pre-existing abdominal pain (5), Constipation (3), Gastrointestinal reaction (2), Nausea (2)	45
	Allergic reaction	2	Allergic urticaria (2)	
	Skin and its appendage	2	Drug eruption (2)	
	Nervous system	1	Insomnia (1)	
		29	Diarrhea (25), Gastrointestinal discomfort (2)	34

Xiaoer Chiqiao Qingre granule	Digestive system		Nausea and vomiting (2)	
	Liver damage	1	Abnormal liver function (1)	
	Skin and its appendage	1	Minor rash (1)	
	Circulatory system	3	Hyperhidrosis (3)	
Dalitong granule	Digestive system	18	Dull abdominal pain and discomfort (7), Borborygmus (5), Epigastric discomfort (2), Lower abdominal discomfort (2), Abdominal distension and discomfort (1), Diarrhea and watery stool (1)	18
Shenshuaining capsule	Digestive system	7	Nausea (4), Increased stool frequency (2), Constipation (1)	17
	Kidney damage	10	Creatinine doubling or dialysis (6), Hyperkalemia (4)	
Ruyi Jinhuang powder	Skin and its appendage	12	Pruritus (5), local redness (2) Pain (1), Rash (1), Contact Dermatitis (1) Local Itching, Red Rash (2), Local Slight Burning Pain (2)	14
	Allergic reaction	2		
Zhuifeng Tougu pill	Digestive system	6	Gastric and abdominal discomfort (3), Epigastric discomfort (2), Acute gastroenteritis (1)	13
	Allergic reaction	4		
	Skin and its appendage	2	Fixed drug eruption (2)	
	Circulatory system	1	Hypertension (1)	
Tianmeng oral liquid	Circulatory system	6	Chest fever (4), Restlessness (2)	12
	Digestive system	4	Dry mouth (4)	
	Allergic reaction	2	Allergy(2)	
Huoxiang Zhengqi oral liquid	Kidney damage	8	Urine routine test positive (urine protein abnormal, urine red blood cell abnormal, urine glucose) (3), Urinary tract infection (2), Urinary tract infection (2), Renal function abnormal (1)	11
	Liver damage	2	Abnormal liver function (2)	
	Digestive system	1	Positive occult blood in stool (1)	

Hewei Zhixie capsule	Skin and its appendage	5		8
	Digestive system	2		
	Nervous system	1		
Xiaochaihu capsule	Skin and its appendage	2	Rash (2)	4
	Digestive system	1	Nausea and vomiting (1)	
	Other	1	Infections (1)	
Huoxiang Zhengqi Dropping pill	Digestive system	3	Nausea (2)<br>Diarrhea (1)	4
	Allergic reaction	1	Anaphylaxis (1)	
Juhong Tanke liquid	Digestive system	4	Mild abdominal distension (1), Stomach discomfort (1), Mild stomach pain (1), Moderate stomach pain (2)	4
Yishen Huashi granule	Digestive system	2	Dry mouth (1), Bitter taste (1)	3
	Skin and its appendage	1	Rash (1)	
Huoxue Zhitong ointment	Skin and its appendage	3	Itchy skin (3)	3
Xingnao Zaizao capsule	Digestive system	3	Poor appetite, Upset stomach (3)	3
Xinsuning capsule	Digestive system	3	Heartburn (1), Epigastric discomfort, Nausea (2)	3
Fufang Xianzhuli liquid	Respiratory system	1	Asthma attack (1)	2
	Allergic reaction	1	Allergic rash (1)	
Guilong Kechuanning capsule	Allergic reaction	2		2
Xiaoxuan Zhiyun tablet	Digestive system	2	Nausea, Gastrointestinal discomfort (2)	2
Tiaowei Xiaozhi pill	Circulatory system	1	Palpitations (1)	2
	Digestive system	1	Dry mouth (1)	
Baizi Yangxin pill	Circulatory system	1	AV Block (1)	1

Huoxiang Zhengqi Soft capsule	Systemic reaction	1	Disulfiram-like reaction (1)	1
Jiawei Huoxiang Zhengqi Soft capsule	Digestive system	1	Nausea (1)	1
Naoliqing capsule	Digestive system	1	Stomach upset (1)	1

Table S8. System/organ involved and clinical manifestations of ADEs related to CPMs containing diester aconitine metabolites

CPMs containing diester aconitine metabolites	Involved system/organ	Frequency	Clinical manifestations of ADE (cases)	Total
Xiaojin capsule (pill, tablet)	Skin and its appendage	238	Rash, Pruritus, Facial edema (123), Skin reaction (45), Urticaria (32), Papules (31), Drug eruption (3), Skin allergy (1), Refractory rash (1), Acute generalized exanthematous pustulosis (2), Flushing (2)	337
	Digestive system	58	Nausea and vomiting (30), Gastrointestinal discomfort (11), Increased stool frequency, Loose stool (5), Mild gastrointestinal disorder (5), Mild tongue numbness (3), Gastrointestinal reaction (2), Abdominal pain (1), Severe diarrhea (1)	
	Liver and kidney damage	9	Organ damage (5), Severe liver injury (3), Cholestatic hepatitis (1)	
	Allergic reaction	15	Allergic reaction (13), Allergic dermatitis (1), Severe allergic reaction (1)	
	ENT reaction	1	Epistaxis (1)	
	Circulatory system	3	Vexation (2), Dysphoria, palpitation and insomnia (1)	
	Blood system	2	Anemia (2)	
	Systemic reaction	2	Low fever (2)	
	Reproductive system	9	Abnormal menstruation (3), Decreased menstrual bleeding (3), Increased menstrual bleeding (2)<br>Menstrual disorder (1)	
Zhonghua Dieda pill	Digestive system	146	Nausea (47), Abdominal pain (24), Vomiting (23), Diarrhea (8), Stomach discomfort (7), Stomach pain (4), Abdominal discomfort (3), Thirst (3), Digestive system reaction (3). Retching (1), Xerostomia (1), Hematemesis (2), Epigastric discomfort (2), Tongue paralysis (3), Loss of appetite (4), Acid regurgitation (4), Gastric dysfunction (5), Hyperchlorhydria (6), Gastric cavity (7), Heartburn (7), Mucosal ulcer (8)	249

	Skin and its appendage	45	Rash (24), Pruritus (14), Topical skin reaction (4), Maculopapular rash (1), Vesiculosa (1) Erythema multiforme (1)	
	Nervous system	28	Dizziness (17), Hypersensitivity (6), Headache (4), Insomnia (1)	
	Muscle and skeletal system	7	Local numbness (5), local pain (1), pain (1)	
	Circulatory system	10	Palpitation (3), Flushing (1), Redness (1), Congestion of face (1) Fever of face (1) Edema of the whole body (2) Swelling of lower limbs (2) , Sweating due to debility (3)	
	Systemic reaction	3	Discomfort (2), Fatigue (1)	
	Urinary system	2	Nephritis (2)	
	Allergic reaction	3	Allergic reaction (2), Allergic nephritis (1)	
	Respiratory system	2	Breath-holding (1), Shortness of breath (1)	
	ENT reaction	2	Tinnitus (1), Abnormal vision (1)	
	Reproductive system	1	Menorrhagia (1)	
Shangshi Zhitong ointment	Allergic reaction	52	Skin allergy (51), Henoch-Schonlein purpura (1)	57
	Skin and its appendage	5	Local mild skin pruritus (2), Angioedema (1), Bladder foreign body (1), Skin flushing, itching (1)	
Zhenggu liquid	Skin and its appendage	21	Skin redness, Warmth, Itching (5), Red papules (5), Rash pruritus (4), Mild small red rash (3), Skin flushing, itching (2), Burning sensation (2)	30
	Allergic reaction	6	Skin allergy (5), Severe allergic rash (1)	
	Digestive system	2	Mild stomach upset (2)	
	Circulatory system	1	Arrhythmia (1)	
Qufeng Zhitong capsule	Digestive system	29	Nausea, nausea (11), Gastrointestinal discomfort (7), Gastrointestinal reactions (6), Gastrorrhagia (2), Pruritus (2), Rash (1)	29
Fufang Xiatianwu tablet	Digestive system	16	Epigastric discomfort (6), Acid reflux (3), nausea (2), Anorexia (2), Gastrointestinal reaction (2) , Gastrointestinal reaction (1)	27
	Skin and its appendage	7	Rash (4), Itchy Skin (3)	

	Liver and kidney damage	2	Mild elevation of transaminase (1), Liver and kidney function damage (1)	
	Nervous system	2	Dizziness (2)	
Qiangli Tianma Duzhong pill	Digestive system	27	Gastrointestinal symptoms such as epigastric discomfort, abdominal distension and anorexia (27)	27
Fuzi Lizhong pill	Circulatory system	9	Ventricular Tachycardia (7), Arrhythmia (2)	17
	Toxic reaction	4	Poisoning (4)	
	Digestive system	2	Mild Thirst (2)	
	Circulatory system	2	Facial edema (2)	
Wumei pill	Digestive system	12	Dry mouth (4), loose stools (3), dry stools (3), mild nausea (1), intolerance (1)	13
	Circulatory system	1	Dizziness Headache (1)	
Zhuifeng Tougu pill	Digestive system	6	Gastric and abdominal discomfort (3), Epigastric discomfort (2), Acute gastroenteritis (1)	13
	Allergic reaction	4		
	Skin and its appendage	2	Fixed drug eruption (2)	
	Circulatory system	1	Hypertension (1)	
Tongbi capsule	Nervous system	2	Headache (1), Dizziness and fatigue (1)	10
	Skin and its appendage	2	Rash (1), Itchy skin (1)	
	Digestive system	1	Stomach nausea (1)	
	Circulatory system	1	Abnormal ECG (1)	
	Urinary system	1	Urinary occult blood (1)	
	Muscle and skeletal system	1	Low back pain (1)	
	ENT reaction	2	Muchi (2)	
Goupi ointment	Skin and its appendage	6	Rash Pruritus (5), Skin Reaction (1)	10
	Allergic reaction	4	Skin Allergy (4)	
Guyouling Liniment	Skin and its appendage	10	Local skin maculopapular rash (8), Rash (1), Skin flushing (1)	10

Xiaohuoluo pill	Circulatory system	5	Palpitation (3), Arrhythmia (2)	10
	Nervous system	3	Dizziness (3)	
	Toxic reaction	2	Poisoning (2)	
Mugua pill	Circulatory system	5	Arrhythmia (5)	7
	Digestive system	1	Purpuric gastritis (1)	
	Liver damage	1	Liver injury (1)	
Jisheng Shenqi pill	Digestive system	6	Pharyngeal discomfort (4), epigastric discomfort (1), transient diarrhea (1)	6
Sanqi Shangyao tablet	Allergic reaction	3	Anaphylaxis (3)	5
	Circulatory system	2	Arrhythmia (2)	
Tianma pill	Allergic reaction	3	Allergic purpura (2), Allergic urticaria (1)	5
	Skin and its appendage	2	Drug eruption (2)	
Guifu Dihuang pill	Digestive system	3	Dry mouth, Nausea (3)	4
	Urinary system	1	Hematuria (1)	
Shengbai oral liquid	Digestive system	3	Epigastric discomfort (2), mild nausea (1)	3
Fugui Gutong capsule	Liver damage	2	Abnormal liver function (2)	3
	Kidney damage	1	Abnormal urine routine (1)	
Medicinal moxa stick	Allergic reaction	2		2
Shexiang Dieda Fengshi ointment	ENT reaction	1	Subarachnoid hemorrhage (1)	1
Shexiang Zhentong ointment	ENT reaction	1	Transient blindness (1)	1
Tongbi tablet	Circulatory system	1	Palpitation with numbness of lips and tongue (1)	1
Tianhe Zhuifeng ointment	Allergic reaction	1	Skin allergy (1)	1
Shaolin Fengshi Dieda ointment	Toxic reaction	1	Poisoning (1)	1

Fugui Gutong granule	Digestive system	1	Nausea (1)	1
Yixin pill	Digestive system	1	Mild dry mouth (1)	1
